# An evaluation of inbreeding measures using a whole-genome sequenced cattle pedigree

**DOI:** 10.1038/s41437-020-00383-9

**Published:** 2020-11-06

**Authors:** Setegn Worku Alemu, Naveen Kumar Kadri, Chad Harland, Pierre Faux, Carole Charlier, Armando Caballero, Tom Druet

**Affiliations:** 1 Unit of Animal Genomics, GIGA-R & Faculty of Veterinary Medicine, University of Liège, Liège, Belgium; 2 Centro de Investigación Mariña, Departamento de Bioquímica, Genética e Inmunología, Edificio CC Experimentais, Universidade de Vigo, Campus de Vigo, As Lagoas, Marcosende, 36310 Vigo, Spain

**Keywords:** Conservation genomics, Animal breeding, Inbreeding

## Abstract

The estimation of the inbreeding coefficient (*F*) is essential for the study of inbreeding depression (ID) or for the management of populations under conservation. Several methods have been proposed to estimate the realized *F* using genetic markers, but it remains unclear which one should be used. Here we used whole-genome sequence data for 245 individuals from a Holstein cattle pedigree to empirically evaluate which estimators best capture homozygosity at variants causing ID, such as rare deleterious alleles or loci presenting heterozygote advantage and segregating at intermediate frequency. Estimators relying on the correlation between uniting gametes (F_UNI_) or on the genomic relationships (F_GRM_) presented the highest correlations with these variants. However, homozygosity at rare alleles remained poorly captured. A second group of estimators relying on excess homozygosity (F_HOM_), homozygous-by-descent segments (F_HBD_), runs-of-homozygosity (F_ROH_) or on the known genealogy (F_PED_) was better at capturing whole-genome homozygosity, reflecting the consequences of inbreeding on all variants, and for young alleles with low to moderate frequencies (0.10 < . < 0.25). The results indicate that F_UNI_ and F_GRM_ might present a stronger association with ID. However, the situation might be different when recessive deleterious alleles reach higher frequencies, such as in populations with a small effective population size. For locus-specific inbreeding measures or at low marker density, the ranking of the methods can also change as F_HBD_ makes better use of the information from neighboring markers. Finally, we confirmed that genomic measures are in general superior to pedigree-based estimates. In particular, F_PED_ was uncorrelated with locus-specific homozygosity.

## Introduction

Inbreeding results from the mating of related individuals and is associated with negative consequences such as inbreeding depression (ID), the reduction in fitness due to increased homozygosity (Hedrick and Garcia-Dorado 2016). Inbreeding depression is common in livestock species (Leroy 2014) and many recessive disorders associated with increased inbreeding have been identified in intensively selected cattle breeds. There are two main hypotheses to explain ID (Charlesworth and Willis 2009). The first is increased homozygosity at partially recessive deleterious alleles (e.g., Charlesworth and Charlesworth 1999) and the second is reduced heterozygosity (equivalent to increased homozygosity) at loci presenting heterozygote advantage (overdominance). Although Charlesworth (2015) concluded that the second hypothesis might be important in Drosophila, most empirical evidences suggest that the first one might be more important for other species (Charlesworth and Willis 2009; Hedrick 2012). The two proposed mechanisms have distinct consequences on allele frequencies. Purging selection maintains deleterious alleles at low frequency or removes them from the population, so that such alleles are mostly young and segregate at low frequency. In contrast, overdominant alleles are maintained at intermediate frequency due to balancing selection (Charlesworth and Willis 2009).

The inbreeding coefficient (*F*) is a tool for the management of populations and for the study of ID. It also provides information on relatedness among parents, mating systems, population structure and recent demographic events (see, e.g., Caballero 2020, Chap 4). Wright (1922) defined the coefficient of inbreeding in terms of correlations between the parents’ uniting gametes. Malécot (1948) offered an alternative definition based on the probability that two homologous alleles in an individual are identical-by-descent (IBD), i.e., they are copies of an allele from a common ancestor. Pedigree-based estimators of the inbreeding coefficient rely on this definition and require the choice of a reference population (e.g., an earlier generation), often determined by the available genealogy. The choice of this reference generation is somewhat arbitrary as individuals from that generation must be considered unrelated. Genomic inbreeding measures can also be estimated using genetic markers. Several authors concluded that genomic measures are better than pedigree-based estimates (e.g., Keller et al. 2011; Wang 2016), mainly because they provide estimators of the realized inbreeding, are robust to pedigree errors and do not require a genealogy.

Numerous genomic estimators of the inbreeding coefficient have been proposed, and there is no consensus on which is the most appropriate (Goudet et al. 2018). Inbreeding measures can for instance be estimated by maximum likelihood approaches (Milligan 2003; Wang 2007), by methods-of-moment (Ritland 1996; Purcell et al. 2007), from the diagonal elements of a genomic relationship matrix (GRM) (VanRaden 2008), from simple heterozygosity or homozygosity measures (Szulkin et al. 2010; Bjelland et al. 2013), based on genotypic correlations (Ackerman et al. 2017) or from the proportion of the genome within runs-of-homozygosity (ROH) (McQuillan et al. 2008; Ferenčaković et al. 2013). These different estimators of the inbreeding coefficient can be evaluated using different approaches. First, their theoretical properties can be derived, as Yengo et al. (2017) did for bias and standard errors, although this was not possible for all estimators. Alternatively, empirical comparisons between estimators can be performed on real datasets, that have been genotyped at low to moderate density with neutral markers selected on their minor allele frequency (MAF), and without knowledge of the true inbreeding coefficient or relatedness (Santure et al. 2010; Bjelland et al. 2013; Pryce et al. 2014; Zhang et al. 2015a; Goudet et al. 2018; Kardos et al. 2018a). In such cases, the coefficients are often compared to the pedigree-based estimates, although the latter should not be considered as the golden standard (Speed and Balding 2015). Performances of estimators can also be evaluated empirically on real data, by testing which measure presents the highest correlation with fitness traits (Kardos et al. 2016). As such, several authors have used statistical criteria to determine which inbreeding coefficients best fit recorded phenotypes (Grueber et al. 2011; Ferenčaković et al. 2017; Clark et al. 2019). Simulation studies can also be carried out (e.g., Milligan 2003; Keller et al. 2011; Druet and Gautier 2017; Nietlisbach et al. 2019) but they rely on an arbitrary definition of the true inbreeding coefficient and other assumptions that are sometimes unrealistic. For instance, they might assume unlinked loci, absence of selection, random mating, equal parent contributions, non-overlapping generations, homogeneous recombination rates, or a simplified population history, such as a constant effective population size (N_e_) or a single bottleneck. Previous comparisons concluded that the best method varied according to parameters such as the number of markers, the number of alleles, the number of individuals, the relatedness within the population, the mating structure or the intended application (e.g., Milligan 2003; Wang 2011; Goudet et al. 2018).

Whole-genome sequence data might provide a good opportunity to empirically evaluate different inbreeding estimators, since genome-wide heterozygosity can be measured extremely accurately (Kardos et al. 2016). With resequencing data, genotypes are available for almost all variants, including those contributing to ID. In addition, genotypes for markers segregating at all frequencies and for alleles from different functional categories, including deleterious variants or those under balancing selection, are present in the data. Consequently, they offer a complementary empirical strategy to evaluate the properties and accuracies of different inbreeding measures, for applications such as the study of ID or the evaluation of conservation and selection programs. For instance, Yengo et al. (2017) used true genotypes available for more than nine million SNPs to simulate ID and compare inbreeding measures in humans. In such an approach, allele frequencies, linkage disequilibrium (LD) patterns and heterogeneity along the genome matched reality. Some assumptions, however, were still required regarding the architecture of ID, such as the class of variants causing it, or the relationship considered between effect size and allele frequency. Thus, the way in which the phenotypes were simulated and how metrics were evaluated had been subject of debate (Kardos et al. 2018b; Yengo et al. 2018; Nietlisbach et al. 2019). We herein propose to follow a similar empirical strategy to evaluate different inbreeding measures in cattle, a livestock species with a very different demographic history compared to human populations. To that end, we used whole-genome sequence data available for a Dutch Holstein cattle pedigree. Inbreeding coefficients were estimated from subsets of markers and the resequencing data was used to estimate homozygosity at different groups of markers. The latter homozygosity measures could for instance serve as proxies for homozygosity at alleles causing ID. Furthermore, performance evaluations were also realized for inbreeding coefficients estimated for specific positions in the genome. Such locus-specific measures would be useful to identify regions contributing to inbreeding depression (e.g., Pryce et al. 2014) or to manage inbreeding at specific loci.

## Material and methods

### Data

We used whole-genome sequences (WGS) from 245 Dutch Holstein cattle sequenced with a coverage higher than 15x. These corresponded to a set of 145 parents and their 100 sequenced offspring. The animals are part of a pedigree containing 743 individuals and sequenced at variable coverage (Harland et al. 2017). The data processing to generate the final Variant Call Format (VCF) file is described in Kadri et al. (2016). This includes description of DNA extraction, library preparation, reads alignment to the reference genome (Bos Taurus UMD 3.1), base quality calibration, variant calling and variant quality score recalibration. For this recalibration of variant quality, we used a set of trusted SNPs from the BovineHD (Illumina) and Axiom Genome-Wide BOS 1 (Affymetrix) commercial genotyping arrays as reference training set.

We selected 12,735,685 autosomal bi-allelic SNPs based on the variant quality score recalibration procedure from GATK (DePristo et al. 2011). The selected SNPs had a variant quality score above the threshold defined to conserve 97.5% from the variants in the reference training set. The inbreeding coefficients were estimated with a subset of 37,675 SNPs present on the Illumina BovineSNP50 BeadChip. Markers located in putative map errors as defined by Kadri et al. (2016) were excluded.

We extracted a genealogy including all the 743 sequenced individuals and their ancestors. The generated pedigree file contained 12,238 individuals, and the 743 sequenced individuals had on average 99.9, 97.2 and 84.0% known ancestors in their 5th, 8th and 10th pedigree generation, respectively.

### Estimation of inbreeding coefficients

The levels of genomic inbreeding were estimated with several measures and using the set of 37,675 SNPs from the commercial array (248 monomorphic SNPs were additionally filtered out for estimation of genome-wide inbreeding coefficients).

A set of estimators of the inbreeding coefficient were obtained from individual SNP data. The first measure (F_UNI_) was based on the correlation between uniting gametes (Yang et al. 2011) and is equivalent to the method proposed by Li and Horvitz (1953) or Ritland (1996). The second measure (F_GRM_) was obtained from the diagonal elements of the genomic relationship matrix (GRM) computed using the first method proposed by VanRaden (2008). These two measures were estimated with GCTA (Yang et al. 2011). The third measure was the excess of homozygosity (F_HOM_), a moment estimator based on the expected and observed individual heterozygosity, implemented in PLINK (Purcell et al. 2007) and proposed by Li and Horvitz (1953). The fourth measure (F_ML_) was the maximum likelihood estimator from Wang (2007) using genotypes of triad of individuals to estimate the nine condensed IBD states (Jacquard 1974). The method is implemented in COANCESTRY (Wang 2011) and the related R package (Pew et al. 2015).

A second set of estimators of *F* was based on sequences of consecutive homozygous SNPs. Runs of Homozygosity (ROH) were detected with PLINK with the following options: a minimum of 50 SNPs per ROH, at least 1 SNP per 100 Kb, a scanning window of 50 SNPs, a total length > 2 Mb, spacing between successive SNPs <500 Kb and no heterozygous SNPs. These ROH were then used to calculate F_ROH_, defined as the proportion of the genome in ROH (McQuillan et al. 2008). A distinction between different ROH length classes (2–5 Mb, 5–10 and >10) were considered, as described in more detail in Supplementary Text S1. Estimators were also obtained from the proportion of the genome in homozygous-by-descent (HBD) segments (Druet and Gautier 2017), closely related to F_ROH_. A comparison of these two last approaches is available in Solé et al. (2017). A hidden Markov model with four HBD classes with rates equal to 5, 25, 125 and 525 was run with RZooROH (Bertrand et al. 2019). These rates are associated to the length of HBD segments in each HBD class: the expected length of HBD segments being equal to 1/R_k_ Morgans, where R_k_ is the rate of the class *k* (corresponding to ancestors present approximately 0.5 × R_k_ generations ago; Hayes et al. 2003). A more complete description of this model can be found in Druet and Gautier (2017) or Solé et al. (2017). The inbreeding coefficient F_HBD_, was estimated as the probability to belong to any of the HBD classes averaged over the whole genome.

In addition to genomic measures, we also estimated the inbreeding coefficient based on the pedigree data (F_PED_; Wright 1922), including a distinction between recent inbreeding, from contributions of ancestors present in the last five generations of the pedigree, and ancient inbreeding, from earlier contributors (see Supplementary Text S1).

### Properties of inbreeding coefficients

The inbreeding coefficient has been defined in terms of correlations between the parents’ uniting gametes by Wright (1922) and as the probability that two homologous alleles in an individual are IBD by Malécot (1948). It also measures the fraction by which the heterozygosity has been reduced due to inbreeding (Crow and Kimura 1970). The different inbreeding coefficients used in the present study match often more closely to one of these definitions. For instance, F_UNI_ is directly related to the definition of Wright (1922), F_PED_ fits that from Malécot (1948) and F_HOM_ measures the heterozygosity reduction. Different estimators thus have different properties, some of which are summarized below. When defined as an IBD probability, estimators must range between 0 and 1 and F_PED_, F_ROH_, F_HBD_ and F_ML_ fall in that category. The other measures, F_UNI_, F_GRM_ and F_HOM_, can take negative values and behave more like correlations (e.g., Wang 2014). A base population where individuals are considered unrelated must also be defined when relying on IBD probabilities. For F_PED_, this corresponds obviously to the founders of the pedigree, whereas for F_HBD_ and F_ROH_, the base population depends on the shortest identified HBD segments as their size is related to the number of generations to the common ancestor. For other coefficients, the base population is indirectly defined by the set of individuals used to estimate the allele frequencies (e.g., Wang 2014). Interestingly, F_HOM_ and F_ROH_ weight all alleles equally, whereas F_UNI_ and F_GRM_, two methods relying on correlations or covariances between genetic effects, give more weight to homozygosity at rare alleles (VanRaden 2008; Keller et al. 2011). Allele frequencies are used differently by F_ML_ and F_HBD_, which rely on the probabilities to observe genotypes conditionally on *F* and Hardy-Weinberg proportions. In both cases, genotypes come from a mixture of two distributions (autozygous *vs* allozygous) and *F* is estimated as the value maximizing the likelihood function. We show that F_HBD_ and F_ML_ are indeed equivalent when the SNPs are considered independent in F_HBD_ (see Supplementary Text S2). Independence between SNPs would correspond to homozygosity-by-descent resulting from very distant ancestors separated by many generations of recombination. Hardy-Weinberg proportions are also used in F_HOM_ to estimate the expected total number of homozygous genotypes. Finally, F_HBD_ and F_ROH_ exploit information from neighboring SNPs, by identifying sequences of homozygous markers. The length of these homozygous stretches is informative about the number of generations to the common ancestor. These estimators also provide the ability to estimate locus-specific inbreeding coefficients. Overall, although the different metrics share some properties, their connections remain relatively complex.

### SNP annotation

To evaluate the properties of different inbreeding coefficients, we compared them for different groups of SNPs from the WGS data. Therefore, we started by classifying the SNPs according to different criteria mainly related with their putative deleteriousness, such as their frequency, age and predicted functional effect.

#### Marker allele frequency

Allele frequency (AF) was selected as a criterion since it is linked to the age of the alleles and to their possible selection coefficient, i.e., their deleterious effect.

#### Age of alleles

Deleterious alleles are expected to be young since purifying selection eliminates them relatively rapidly. Unfortunately, we did not know the true age of the alleles identified in our dataset, yet some indicators were available. First, allele frequency can be utilized as a proxy for relative allele age (Kelleher et al. 2019). Secondly, alleles observed in multiple populations (or breeds) can be assumed to be on average older than alleles observed only in our Holstein pedigree. Thus, alternate alleles not observed in a sample of 50 whole-genome sequenced Belgian Blue Beef cattle used in Charlier et al. (2016), hereafter referred to as ‘private alleles’, allowed us to enrich our set of variants in young alleles.

To validate these hypotheses, we used the approach of Albers and McVean (2020) for dating genomic variants implemented in GEVA (Genealogical Estimation of Variant Age). We first phased variants from *Bos taurus* autosome (BTA) 25 with Beagle 4.0 (Browning and Browning 2007) using the pedigree option. We then ran GEVA and relied on the recombination clock to estimate the age of alleles.

#### Functional annotation

Deleterious or beneficial variants are more likely to be coding or regulatory variants. Therefore, the VCF file was annotated into different functional categories using Variant Effect Predictor (VEP) (McLaren et al. 2016). VEP predicts consequences of variants on protein sequence and uses Sorting Intolerant From Tolerant (SIFT) scores (Ng and Henikoff 2003) to determine which amino acid substitutions are deleterious or tolerated. Three classes of SNPs were then created using this information: synonymous variants, tolerated missense variants and deleterious missense variants. Variants classified as ‘low confidence’ by VEP were excluded from the analysis.

### Empirical evaluation of the properties of inbreeding coefficients using metrics computed from WGS data

We compared different measures of inbreeding estimated with the 37,675 array-like SNPs with homozygosity measured at different groups of alleles from the WGS data including 12,735,685 autosomal SNPs. These latter homozygosity measures were used to mimic homozygosity at alleles causing ID or the impact of inbreeding on whole-genome homozygosity. Correlations between different estimators and these homozygosity scores were used to evaluate the performances of the different methods. The standard errors of these correlations were obtained using the Fisher transformation.

#### Homozygosity for different allele frequency groups

We computed homozygosity of alternate alleles grouped in different frequency classes (e.g., 0.0–0.05, 0.05–0.10, etc.) to understand how efficiently inbreeding coefficients captured homozygosity in these different classes. Similarly, we estimated marker homozygosity (i.e., homozygosity at both reference and alternate alleles) per class of MAF. Note that this measure also reflects heterozygosity, as both measures sum to one. All these metrics helped us to compare the general properties of inbreeding coefficients, but also their association with subsets of SNPs potentially associated with ID. For instance, homozygosity at low frequency alleles would be related to the homozygosity at partially recessive detrimental alleles, whereas heterozygosity at intermediate frequency alleles would be associated with the heterozygosity at overdominant alleles.

#### Whole-genome sequence homozygosity

We next considered the total WGS homozygosity as another metric, capturing the genome-wide impact of inbreeding. Inbreeding increases homozygosity at all loci of the genome simultaneously (e.g., Szulkin et al. 2010; Wang 2014). Consequently, the inbreeding coefficient can be measured as the fraction by which heterozygosity has been reduced (Crow and Kimura 1970), and WGS homozygosity has been suggested as a measure to empirically evaluate different inbreeding estimators (Kardos et al. 2016).

#### Homozygous mutation load (HML)

Following Keller et al. (2011), we counted the number of rare or low-frequency alleles that were homozygous per individual, considered to be a proxy for ID. We computed HML at different allele frequency (AF) thresholds (0.05, 0.10 to 0.15) to determine whether results were sensitive to frequency of the alleles included in the HML score.

A weighted HML (wHML) was also computed using the inverse of allele frequency as weights, as deleterious effects are expected to be stronger for rarer alleles (e.g., Yengo et al. 2017). HML scores were also computed specifically for non-synonymous, tolerated and deleterious missense variants.

#### Regional scores (locus-specific)

To study the properties of regional inbreeding coefficients at a specific locus, we estimated regional homozygosity and regional HML scores using all SNPs present in non-overlapping 1 Mb windows, and compared them with regional measures of the inbreeding coefficient. F_UNI_ or F_GRM_ were computed using only the markers from the Illumina BovineSNP50 BeadChip present in the respective 1 Mb window. For F_HBD_ and F_ROH_, we averaged the HBD probabilities and the ROH status (0/1), respectively, from SNPs in the window. For regional homozygosity, windows with less than 5 SNPs on the 50 K array were excluded from the analysis, whereas for regional HML, windows with less than 2000 variants from the WGS data, less than 5 SNPs on the 50 K array or less than 8 individuals with a non-zero score, were excluded.

## Results

### Inbreeding measures

Descriptive statistics of the estimated inbreeding coefficients are reported in Table S1. F_ML_, F_HBD_, F_ROH_ and F_PED_ values were always positive. With F_ML_, 81 out of 145 parents had a null inbreeding coefficient whereas no null values were reported for F_PED_, F_HBD_ or F_ROH_. With the exception of F_ML_, genomic measures presented higher variances than F_PED_, with the largest values observed for F_GRM_ followed by F_HOM_ and F_UNI_. The correlations between all measures are available in Table S2. The HBD-based measure was highly correlated with F_ROH_ (0.95) and with the excess of homozygosity (F_HOM_) measure (0.96). Their correlations with F_PED_ were relatively high, equal to 0.76, 0.77 and 0.83 for F_HBD_, F_HOM_ and F_ROH_, respectively. The correlation between F_UNI_ and F_GRM_ was also strong (*r* = 0.88). The correlation between F_UNI_ and F_ML_ was 0.76, but increased to 0.96 when only the individuals with F_ML_ > 0 were considered (Supplementary Fig. S1). Both measures had high or moderate correlations with all other measures. Measures of F_PED_, F_ROH_ and F_HBD_ considering all pedigreed generations and all fragment lengths showed larger correlation with the other *F* estimators than measures assuming only recent/ancient generations, or short/long fragments (Table S2). Thus, only full measures will be considered in the following, where F_HBD-525_ will be referred to as F_HBD_. Additional details on the results obtained with these partitioned *F* measures are given in Supplementary Text S1 and Supplementary Figs S2-5.

### Age of alleles

Using the software GEVA we predicted the age of 231,111 alternate alleles located on BTA25 using the recombination clock. The Time to the Most Recent Common Ancestor (TMRCA) was estimated in generations and represented a relative measure that allowed us to compare categories of alleles. Private alternate alleles had clearly lower average TMRCA (462) than alternate alleles in general, private alleles included (1758). Table 1 provides the average TMRCA for both types of alleles classified according to their allele frequency. Alleles segregating at lower frequency were younger and more so if they were not present in the Belgian Blue cattle sample (i.e., private). Interestingly, private variants were enriched in low frequency alleles, with very few alleles having a frequency >0.30. In summary these results confirmed that private alleles segregating at low frequency were enriched in young alleles.

**Table 1:** Average age of alternate alleles from BTA25, expressed in Time to the Most Recent Common Ancestor (TMRCA), estimated with GEVA and using the recombination clock.

	Alternate alleles		Private alternate alleles	
Allele frequency	Number of alleles	Average TMRCA	Number of alleles	Average TMRCA
0 <. ≤ 0.05	59,943	604	19,108	361
0.05 <. ≤ 0.15	50,596	1284	6136	564
0.15 <. ≤ 0.30	43,931	1820	1633	772
.> 0.30	76,281	2946	180	4851

The time is measured in generations. Private alleles refer to alleles absent from a whole-genome sequence data sample available for another breed (Belgian Blue cattle). Private alleles are selected to enrich the set of SNPs in young alleles.

### Genome-wide comparisons of estimated inbreeding coefficients

#### Correlations with homozygosity measured in different allele frequency (AF) classes

**Fig. 1:**
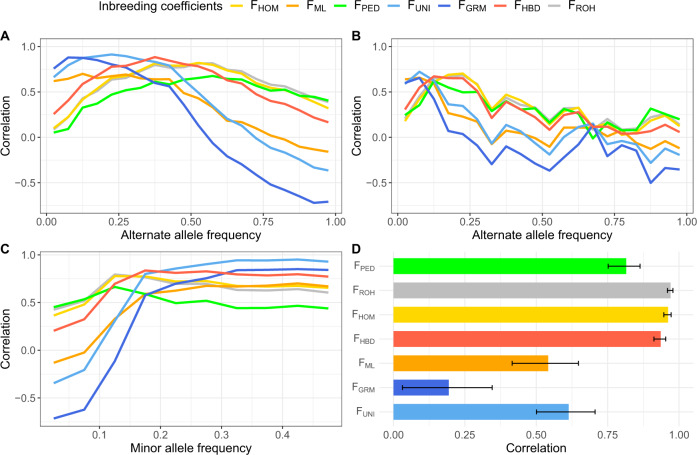
Correlation coefficients between individual inbreeding measures estimated with 37,675 SNPs and scores obtained from the whole-genome sequence data in 145 individuals. **a** Correlation with homozygosity at alternate alleles grouped according to their allele frequency. **b** Correlation with homozygosity at private alleles (young alleles) grouped according to their allele frequency. **c** Correlation with global marker homozygosity (counted at both reference and alternate alleles) as a function of minor allele frequency. **d** Correlation with whole-genome homozygosity. The error bars represent the 95% confidence intervals.

The correlations between the inbreeding coefficients and the homozygosity at alternate alleles grouped according to their frequency (20 classes) are plotted in Fig. 1a. For the least frequent alleles, correlations ranged from as low as 0.05 (F_PED_) to 0.76 (F_GRM_). Alleles with slightly larger frequencies (from 0.05 to 0.15) presented higher correlations with all metrics, indicating that the lowest frequency alleles might be more difficult to capture with inbreeding coefficients. For allele frequencies below 0.25, the two methods giving more weights to rare alleles (F_UNI_ and F_GRM_) performed the best followed by likelihood-based methods (F_ML_ and F_HBD_). Metrics giving equal weight to all alleles such as F_HOM_ and F_ROH_ were less efficient for rare alleles, but better for homozygosity at frequent alleles for which F_UNI_, F_GRM_ and F_ML_ were clearly less useful.

The pedigree-based measure (F_PED_) achieved the lowest correlations and presented patterns similar to F_HOM_ and F_ROH_, estimators relying on whole-genome homozygosity. For instance, all these estimators presented lower correlations when homozygosity was estimated using rare alleles.

#### Correlations with homozygosity at private alleles

When homozygosity was computed using private alleles (enriched in younger alleles), F_UNI_, F_GRM_ and F_ML_ were still the best estimators to capture homozygosity at rare alleles (AF < 0.10), particularly for the lowest frequency class (Fig. 1b), but these correlations were lower than in the previous section. Conversely, the correlations increased for other inbreeding measures, which became more efficient when homozygosity was measured specifically at private alleles. Consequently, the methods presented smaller differences in terms of correlations. For alleles with frequencies ranging from 0.10 to 0.25, F_HBD_, F_HOM_, F_ROH_ and F_PED_ were even more efficient than F_UNI_, F_GRM_ and F_ML_ for which correlations were strongly reduced. Finally, for the class of variants with an AF > 0.25, the small number of private alleles reaching these frequencies reduced the reliability of the analysis.

#### Correlations with marker homozygosity measured in different MAF classes

Correlations between inbreeding coefficients and marker homozygosity are in line with observations for allele homozygosity metrics (Fig. 1c). Indeed, marker homozygosity at SNPs with low MAF results mainly from homozygosity at frequent alleles. Consequently, methods that captured well homozygosity at frequent alleles (F_HOM_, F_ROH_ and F_PED_) had the strongest correlations with marker homozygosity at SNPs with low MAF. Conversely, F_UNI_, F_GRM_ and F_HBD_ performed better when MAF was higher than 0.25. Overall, most inbreeding coefficients were better at capturing marker homozygosity for alleles segregating at intermediate frequency (MAF > 0.15) than homozygosity at private and rare alleles (AF < 0.15).

#### Correlations with whole-genome homozygosity

When whole-genome homozygosity was estimated for all alleles (Fig. 1d, Supplementary Fig. S6), irrespective of their MAF or age, F_ROH_ and F_HOM_ presented the highest correlation (0.97 and 0.96, respectively), closely followed by F_HBD_ (0.94). Interestingly, F_PED_ was also highly correlated (0.81), even more than the remaining genomic measures, which give more weight to rare alleles, while F_GRM_ presented a relatively weak correlation with this score (0.19).

#### Correlations with homozygous mutations load (HML)

**Fig. 2:**
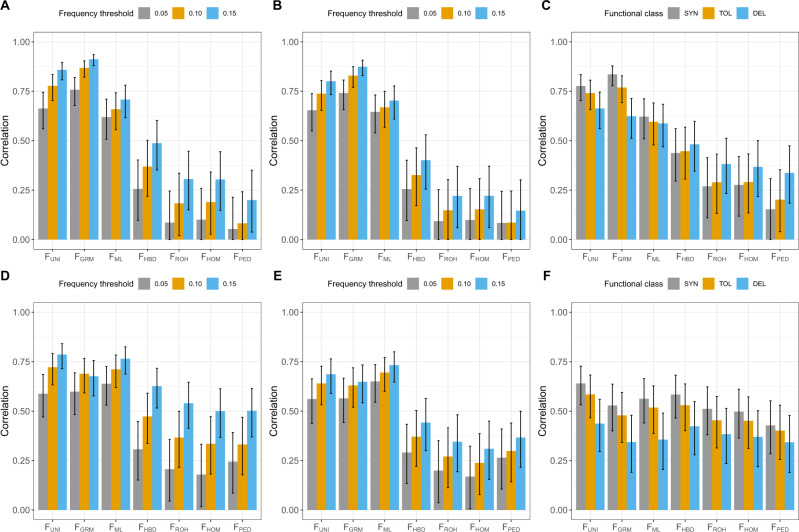
Correlation coefficients between individual inbreeding measures estimated with 37,675 SNPs and homozygous mutation load (HML) obtained from the whole-genome sequence data in 145 individuals. HML was computed using alternate (**a**, **b**, **c**) and private alternate (**d**, **e**, **f**) alleles. **a** and **d** Correlation with HML estimated with allele frequency thresholds of 0.05, 0.10 and 0.15. **b** and **e** Correlation with weighted HML estimated with allele frequency thresholds of 0.05, 0.10 and 0.15. **c** and **f** Correlation with HML estimated with synonymous (SYN), tolerated (TOL) and deleterious (DEL) missense variants and using an allele frequency threshold of 0.15. The error bars represent the 95% confidence intervals and are truncated at 0.

Methods giving more weight to rare alleles such as F_UNI_ and F_GRM_, better captured HML (Fig. 2a, Supplementary Figs S7–9) in agreement with their better correlation with homozygosity at rare alleles. The differences with other estimators were larger for lower AF thresholds. We subsequently weighted alleles by the inverse of their frequency, as rare alleles are more likely to have strong deleterious effects (e.g., Yengo et al. 2017). In that case, correlations varied little for the lowest frequency threshold (0.05), whereas for higher thresholds the correlations were somehow reduced as expected (Fig. 2b). The HML was then computed for synonymous, missense tolerated and missense deleterious variants with an AF threshold set at 0.15 (Fig. 2c, Supplementary Figs S10–12). Alleles in the most damaging classes were less frequent. Correlations obtained with metrics such as F_UNI_, F_GRM_ and F_ML_ decreased, more so for more deleterious classes. Interestingly, the other measures had the opposite behavior: higher correlations for these specific classes than for general HML and better performances for more deleterious alleles. Nevertheless, their performance was still below that from the first group of methods. F_ROH_ had correlations similar to those obtained with F_HOM_.

As deleterious alleles are expected to be rare and young, we re-estimated the HML and wHML using only private alleles that are enriched in young alleles. As observed before, F_UNI_ and F_GRM_ had lower correlations with private alleles, whereas the other methods performed better (Fig. 2d–e, Supplementary Figs S13–15). As a result, differences between methods were smaller and F_ML_ performed better than F_GRM_. Interestingly, all inbreeding coefficients had correlations higher than 0.50 with AF threshold set at 0.15. When HML was computed with synonymous or missense variants, F_UNI_ still presented the highest correlations but F_HBD_ was now second, for all three sets of variants (Fig. 2f, Supplementary Figs S16–18). With private alleles, homozygosity at more deleterious alleles was more difficult to capture irrespective of the method. As before, correlations obtained with F_UNI_, F_GRM_ and F_ML_ dropped when considering only the variants in coding regions whereas correlations with other metrics were less impacted. As a result, smaller differences were observed between methods, in particular when HML was computed with deleterious missense variants only. With these HML scores derived from private alleles, F_ROH_ performed better than F_HOM_. Note that when private alleles with specific annotations were used, HML scores were derived from fewer variants. Therefore, these correlations should be interpreted cautiously.

### Comparisons with regional scores

Inbreeding measures were also evaluated for their association with regional scores estimated in 1 Mb non-overlapping windows (see methods). F_ML_ was excluded from the comparisons due to its long running times and because it did not perform best in genome-wide comparisons.

**Fig. 3:**
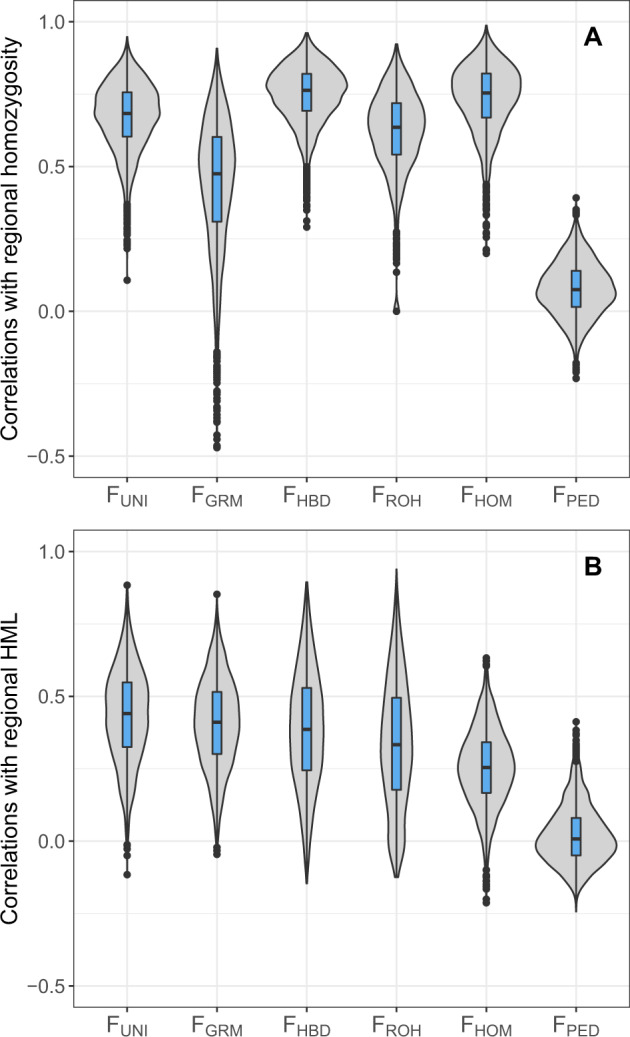
Correlation coefficients between individual regional inbreeding measures and regional scores in 1 Mb windows computed from the whole-genome sequence data in 145 individuals. The regional inbreeding coefficients were estimated only with markers present among the 37,675 SNPs from the bovine genotyping array (see Methods for more details). The correlations for ~2500 windows are presented as a violin plot combined with an inner boxplot. **a** Correlation with regional homozygosity. **b** Correlation with regional homozygous mutation load (HML).

We started by computing regional homozygosity measured at all alleles irrespective of their frequency (Fig. 3a). When averaged over all windows, correlations with genomic inbreeding coefficients were relatively high for F_HBD_ (0.75), F_HOM_ (0.74), F_UNI_ (0.67) and F_ROH_ (0.62) but somewhat lower with F_GRM_ (0.44). Pedigree-based estimators were clearly below all genomic measures with an average correlation close to zero (0.08). There was nevertheless considerable variation between regions of the genome, in particular with F_GRM_ (Fig. 3a).

Regional HML was then computed using alternate alleles with AF ≤ 0.15 (Fig. 3b). Correlations between local inbreeding measures and regional HML were lower than those obtained with whole-genome HML scores and showed a larger variation. For instance, they ranged from −0.12 to 0.88 for F_UNI_. On average, F_UNI_ performed best (0.43), followed by F_GRM_ (0.41), F_HBD_ (0.39), F_ROH_ (0.34) and F_HOM_ (0.25), whereas F_PED_ had almost null average correlations (0.02). The ranking of the methods changed however from window to window.

## Discussion

We utilized cattle whole-genome sequence data to empirically evaluate different estimators of the inbreeding coefficient. This sample represents a population with small N_e_ and under intense selection. It brings therefore complementary information to studies relying on populations with large N_e_, such as humans (e.g., Yengo et al. 2017). It is informative for agricultural species but also for wild species with small N_e_, including for populations in conservation programs. Our results must be interpreted cautiously, in particular for the rarest alleles, as the sample size was relatively small. Nevertheless, this approach revealed some properties of the inbreeding coefficient estimators. A first group of methods that give higher weight to homozygosity at rare alleles, including F_UNI_ and F_GRM_, presented the strongest correlations with both genome homozygosity at rare alleles and marker homozygosity at SNPs with moderate to high MAF. A second group of metrics based on the number of homozygous SNPs that give equal weights to all alleles, including F_HOM_ and F_ROH_, achieved the highest correlations with whole-genome homozygosity, but were less efficient to capture homozygosity at rare alleles. When homozygosity was measured for private sets of alleles that were shown to be enriched in young alleles, the performance of the latter measures improved whereas it decreased for the first group of estimators. Interestingly, the properties observed for F_PED_ matched those of the second group. The first group of methods relies on correlations between parental gametes (F_UNI_) or variances of genotypes within an individual (F_GRM_) and better fits the definition of the inbreeding coefficient in terms of correlation proposed by Wright (1922). Conversely, the second group behaving similar to F_PED_ would correspond to the definition by Malécot (1948), relying on the probability that two homologous alleles in an individual are IBD (without imposing any constraint on locus position or on allele frequency). Indeed, they performed better when alleles are young (i.e., more likely to be IBD) and measure the increased proportion of homozygosity (correlated with the increased proportion of autozygosity) at all variants irrespective of their frequency. The last two measures, F_ML_ and F_HBD_, both relying on likelihood maximization (see Methods), presented intermediate properties. We observed that F_ML_ was highly correlated with F_UNI_ for positive inbreeding coefficients (Supplementary Fig. S1) and thus behaved in a manner similar to the first group. In contrast, F_HBD_ was closer to the properties of the second group. Although F_HBD_ uses allele frequencies to compute HBD probabilities, homozygous genotypes that are in long HBD segments receive the same weight irrespective of their AF, as it occurs with F_ROH_ or F_HOM_.

Our approach can also be used to investigate other aspects related to inbreeding coefficients. For instance, we also studied the ability from different methods to work regionally. Such locus-specific estimators could be useful for performing homozygosity mapping experiments to identify regions associated with recessive diseases or ID (Abney et al. 2002; Leutenegger et al. 2006). Similarly, the approach allows the study of the properties of inbreeding coefficients estimated at lower marker density (Supplementary Text S3). Robustness at low marker density is important for applications in agricultural species, where such low-density arrays are sometimes used to reduce genotyping costs, but also for non-model species where high-density arrays might not be available. For both applications, the ranking between methods and their properties remained in line with the high-density results (Supplementary Figs S19–21). As expected, regional homozygosity or HML were more difficult to capture than genome-wide scores. With fewer markers, correlations were also lower, but this reduction remained limited for most methods. Interestingly, F_HBD_ was still efficient at low marker density and appeared to be a good compromise in that case, particularly for regional scores (Supplementary Fig. S21). The method uses local information from neighboring SNPs and the genetic map in a probabilistic framework that accounts for uncertainty; two important elements at low density. With the same approach, properties of recent and ancient inbreeding could also be revealed. For instance, estimators obtained with long *versus* short ROH or using recent *versus* ancient pedigree generations can be compared (Supplementary Text S1). In both cases, inbreeding coefficients using all ROH or all pedigree-generations presented the highest correlations with homozygosity measures (Supplementary Figs S2–3). Nevertheless, the longest ROH (>5 Mb) or the five last pedigree generations accounted for most of the variation between individual inbreeding levels (Supplementary Table S1). Associated estimators performed relatively well, even when ROH were restricted to >10 Mb. Conversely, inbreeding coefficients associated with short ROH (<5 Mb) or with more ancient pedigree generations presented limited variation. Likewise, HBD-measures including all HBD segments better captured homozygosity at rare alleles or HML than related measures considering only the longest segments associated with recent ancestors (Supplementary Figs S4–5). Finally, we also investigated the properties of inbreeding coefficients predicted in offspring thanks to parental genotypes (Supplementary Text S4). Such predictions are important to manage inbreeding levels in livestock species or in conservation programs. With these predicted values, correlations with scores computed from the WGS data were lower than when the inbreeding coefficient was estimated using the genotypes from the individual, as expected (Supplementary Figs S22–24). The same dichotomy between methods predicting well homozygosity at rare alleles and those capturing better whole-genome homozygosity was observed.

The properties highlighted by our empirical approach can also contribute to understand properties from heterozygosity-fitness correlation (HFC) approaches (e.g., Pemberton 2004; Szulkin et al. 2010). The absence of HFC in certain studies has generated debate in the past (David 1998; Pemberton 2004; Szulkin et al. 2010). Several hypotheses have previously been proposed to explain this observation (e.g., David 1998; Slate and Pemberton 2002; Szulkin et al. 2010). For instance, it was postulated that heterozygosity at a few markers (most often micro-satellites) might not capture heterozygosity at other variants, in particular those causing ID (e.g., Balloux et al. 2004; Grueber et al. 2011). It was recommended to use identity disequilibrium measures (Balloux et al. 2004; Slate et al. 2004) to evaluate the correlation between homozygosity at different loci and to assess whether marker heterozygosity was expected to capture differences in genome-wide heterozygosity levels resulting from inbreeding. Here, we observed that with 6000 SNPs (Supplementary Text S3, Supplementary Fig. S19) the genome-wide homozygosity was highly correlated with inbreeding coefficients related to marker homozygosity (F_ROH_, F_HOM_, F_HBD_). However, the homozygosity at rare (deleterious) alleles proved more difficult to capture. Therefore, the correlation with fitness or ID might still be low even when identity disequilibrium is high, for instance if identity disequilibrium is measured among frequent alleles and does not reflect correlation with rare deleterious alleles. Several studies also reported that pedigree measures might present higher correlations with fitness than marker heterozygosity, and recommended F_PED_ as the inbreeding measure to use (e.g., Pemberton 2004; Grueber et al. 2011; Nietlisbach et al. 2017). These results were however most often obtained with relatively few markers (e.g., Grueber et al. 2011; Nietlisbach et al. 2017) and several authors subsequently stated that genomic measures were superior to pedigree-based estimators (e.g., Keller et al. 2011; Wang 2016). Here, we confirm that marker-based inbreeding coefficients performed better than pedigree-based ones, in particular for regional scores that had almost null correlations with F_PED_.

Inbreeding depression is mainly caused by an accumulation of partially recessive deleterious mutations (Charlesworth and Charlesworth 1999) which, in general, are young and remain at low frequency (e.g., Pritchard 2001). Accordingly, HML has been proposed by Keller et al. (2011) as a proxy for ID. They showed in their study that the homozygosity at alleles with a frequency below 0.05 was indeed similar to homozygosity at recessive deleterious alleles. However, the optimal AF threshold depends on the population demographic history and its N_e_. When N_e_ is low, as for livestock species, domestic animals or endangered species, alleles with larger selection coefficients can remain effectively neutral (as long as N_e_ s ≪ 1) and deleterious alleles can reach higher frequencies compared to human populations (Kimura 1983). Since selection is less effective in small populations, mildly deleterious mutation can accumulate (Keller and Waller 2002) and even become fixed (Frankham 1995). When N_e_ ≤ 100, as in several cattle breeds, mildly deleterious variants might reach frequencies around 0.15. Furthermore, mildly deleterious alleles might also segregate at high frequencies as a result of population bottlenecks experienced during domestication or breed creation, and as a result of artificial selection for linked favorable variants, through genetic hitch-hiking (see Bosse et al. 2019). As an illustration, genetic variants causing recessive defects reached frequencies above 0.10, and even higher in the most extreme cases, in Belgian Blue cattle (Fasquelle et al. 2009; Sartelet et al. 2012; Druet et al. 2014; Charlier et al. 2016), but these alleles provided a potential heterozygous advantage. In the present study, F_UNI_, F_GRM_ and F_ML_ captured HML better than other metrics, more so for rare alleles, suggesting that these methods could be more suited to estimate ID and to avoid fitness reduction associated with inbreeding in mating designs. When HML was computed with private alleles enriched for young alleles, the second group of estimators started to behave better and differences between methods were smaller. In particular, when HML was estimated for young and deleterious alleles with properties similar to those of variants causing ID, F_HBD_, F_ROH_ and F_HOM_ had higher correlations than F_GRM_ or F_ML_. Among the methods from the second group, F_HBD_ performed best, notably for regional scores and estimations at lower marker density. In humans, long ROH are enriched in homozygous deleterious alleles (Szpiech et al. 2013) whereas Zhang et al. (2015b) observed the opposite in cattle. Here, we show that both in terms of estimations or predictions, higher correlations with homozygosity at rare and young deleterious variants are obtained when also including shorter HBD segments or ROH (Supplementary Text S1). This is in agreement with recommendations from Kardos et al. (2018a, 2018b) and indicates that at least some of the deleterious variants are present in short HBD segments. However, it is important to keep in mind that HML is an imperfect proxy of ID and that all these correlations with HML must be interpreted cautiously. It is not known which variants are truly deleterious and whether alleles have favorable or negative effects. Ideally, we should use the variants causing ID, weighted by their effect. Finally, note that HML, and more particularly regional HML, could also somehow be related to the d² metric, which measures the distance between microsatellites alleles to capture their time of coalescence (Coulson et al. 1998). The number of homozygous SNPs reflects to a certain extent how closely related the uniting gametes were for that locus.

Overall, our empirical results illustrate that the best inbreeding coefficient estimator might depend on the frequency, age and effect size of alleles contributing to inbreeding depression and the population demographic history. Even for evaluating ID caused by recessive deleterious alleles, the present and past effective population size and the size of the allele effects will result in a different distribution of AF. Although F_UNI_ and F_GRM_ presented high correlations with homozygosity at rare alleles, other metrics might perform better for other groups of alleles. In case the contribution from heterozygosity at loci presenting heterozygous advantage to ID is important, as suggested by Charlesworth (2015), inbreeding coefficients should capture homozygosity at these loci segregating at intermediate frequency (see Fig. 1c). Overall, inbreeding coefficients presented higher correlations with homozygosity at such loci than with homozygosity at rare alleles. Therefore, in that scenario, inbreeding coefficients would present higher correlations with ID, and F_UNI_ or F_HBD_ would perform best as they had the highest correlations with homozygosity at the target loci (Fig. 1c). The fact that different metrics capture homozygosity at SNPs with different properties makes the conclusions from different simulation studies difficult to interpret. Indeed, Yengo et al. (2017) had strong conclusions in favor of F_UNI_ as a preferred measure to estimate ID with human data, whereas Keller et al. (2011) or Nietlisbach et al. (2019) presented results in favor of F_ROH_. However, phenotypes were simulated with different approaches and in populations with different structures. More recently, Caballero et al. (2020) have shown that the results of these papers are not contradictory. In scenarios of large population sizes, such as in human populations, F_UNI_ can be an appropriate inbreeding measure to estimate ID, whereas in scenarios of small population sizes, F_ROH_ may be more appropriate. Therefore, the inbreeding coefficient achieving the highest correlation with ID might differ according to the scenarios and populations considered.

The optimal inbreeding coefficient estimator varies also according to the intended application. When the inbreeding coefficient is used to measure the heterozygosity reduction at all alleles, irrespective of their frequencies or their age, the use of the second group of methods, which are more related to the proportions of autozygous genotypes (F_HBD_, F_HOM_, F_ROH_ and F_PED_) is recommended. This information is important when the objective is to determine the extinction risk of a population, to assess whether a conservation program is efficiently implemented, to understand the recent demographic history from a population, or to estimate the effective population size. Similarly, these measures are useful to investigate mating systems in a population or to identify consanguineous matings. They might also be used to minimize inbreeding in small captive populations and to maintain diversity at all variants. In this group, F_HBD_ (or F_ROH_) performed best and should be preferred to F_HOM_ or F_PED_, in agreement with Keller et al. (2011). These measures are, in addition, easier to interpret as they have positive values and represent autozygosity accumulated relative to a base population. F_HBD_ also behaves well at lower marker densities and can be used to estimate locus-specific inbreeding coefficients or to perform homozygosity mapping experiments to identify regions associated with recessive diseases or ID (Abney et al. 2002; Leutenegger et al. 2006).

The results we have reported present limitations since they relied on some approximations. In particular, our sample size was relatively modest, and this could influence some results. It contained healthy adult animals and did not include individuals that suffered problems earlier in life. Ideally, such an evaluation of inbreeding measures should be performed on larger samples of unselected animals. Measuring directly inbreeding depression in a large cohort of individuals as done by Yengo et al. (2017) would represent a complementary and valuable empirical evaluation of different inbreeding coefficients. Indeed, Szulkin et al. (2010) and Kardos et al. (2016) suggested that the most precise inbreeding measures should present the strongest association with ID.

## Conclusions

Using an empirical approach relying on whole-genome sequence data from a small cattle pedigree, we studied the properties from different inbreeding coefficients. For instance, F_UNI_ was shown to have the highest correlations with rare alleles and might therefore present a strong association with ID when it results from the action of rare recessive deleterious alleles. Nevertheless, ID might remain difficult to capture when associated with rare missense variants. For locus-specific inbreeding measures, the ranking of the methods might change since F_HBD_ makes better use of the information from neighboring markers. Measures related to homozygosity (F_HBD_, F_ROH_ or F_HOM_) were more efficient to capture the proportion of the genome that is IBD, irrespective of allele frequency or age of alleles. Since F_UNI_ and F_HBD_/F_ROH_ present complementary properties, they might both be used when testing for ID. Finally, we confirmed that genomic measures are superior to pedigree-based estimates. In particular, F_PED_ was uncorrelated with locus-specific scores.

## Supplementary information

Supplementary Material

## Data Availability

The genotypes used in the present study are available from the Dryad Digital Repository 10.5061/dryad.vx0k6djq8. The repository also contains the annotation of all the variants and the genotypes for subset of markers present of the bovine genotyping arrays. Original VCFs files are also available at https://www.ebi.ac.uk/ena/browser/view/PRJEB38336, under the name BPWG.vcf.gz.
