# Supplementary material for: An evaluation of inbreeding measures using a whole-genome sequenced cattle pedigree
Source: Heredity (Edinb). 2020 Nov 6;126(3):410–23. doi: 10.1038/s41437-020-00383-9 (PMC8027009; doi:10.1038/s41437-020-00383-9)
Supplement: Supplementary file 1 — Supplementary Material [file 41437_2020_383_MOESM1_ESM.pdf]

Supplementary Text S1

Estimation of inbreeding coefficients associated with recent and ancient inbreeding

Methods

Several methods allow for the computation of inbreeding levels associated with recent or ancient ancestors.

For F_{PED}, we re-estimated the inbreeding coefficients by considering only inbreeding loops of 10 generations or less. The estimator (F_{PED-5G}) corresponds to the contribution of ancestors present in the first five generations of the pedigree. The differences between F_{PED} and F_{PED-5G} represent the inbreeding levels associated with older ancestors and is referred to as $F_{PED-OLD}$.

Length of ROH is related to the number of generations to the common ancestor of the homozygous segment. Therefore, it can also be used to define measures associated with recent or ancient inbreeding. Here, we divided F_{ROH} into short segments of 2 to 5 Mb (F_{ROH-S}), intermediary segments of 5 to 10 Mb (F_{ROH-M}) and segments longer than 10 Mb (F_{ROH-L}). The longer segments are associated with more recent ancestors, although ancestors present 5 generations ago would generate ROH in all three categories. We also computed inbreeding coefficients including all ROH (F_{ROH}) and ROH longer than 5 Mb (F_{ROH-5}).

Similarly, HBD-measures estimated uing only long HBD segments are associated with recent ancestors. Therefore, we defined several HBD-based measures by using subsets of the HBD-classes. As in Solé et al. (2017), the inbreeding coefficient, F_{HBD-T}, was estimated as the probability to belong to any of the HBD classes with a rate $R_k \leq T$ averaged over the whole genome, setting the base population to approximately $0.5 \times T$ generations ago. Here, we computed F_{HBD-25}, $F_{HBD-125}$ and $F_{HBD-525}$. For instance, F_{HBD-25} represents inbreeding associated with recent ancestors, up to approximately 12 generations in the past. Conversely, $F_{HBD-525}$ corresponds to all HBD segments that can be captured with the available marker density and thus, is a measure associated with all ancestors. Note also that $F_{HBD-525}$ is equivalent to F_{HBD} described in the main document.

Results

Descriptive statistics and correlations among inbreeding estimators. With pedigree-based estimators, recent inbreeding (F_{PED-5G}) had higher levels of variation (Supplementary Table S1) and higher correlation (Supplementary Table S2) with other measures, than ancient inbreeding ($F_{PED-OLD}$). F_{PED-5G} was close to F_{PED} ($r = 0.97$) and accounted for most of its variation. Conversely, $F_{PED-OLD}$ presented little variation and low correlations with other inbreeding coefficients.

Among ROH-based estimators using a subset of ROH, F_{ROH-L} presented the largest variation (Supplementary Table S1) and the highest correlation (0.94) with F_{ROH} (including all ROH) and other inbreeding estimators (Supplementary Table S2). Estimators relying on intermediate ROH, of 5 to 10 Mb, presented moderate correlations with other estimators indicating that they partially capture the variation in inbreeding levels. Finally, F_{ROH-S} had the lowest correlations with other inbreeding estimators and presented the lowest levels of variation.

HBD-based measures were highly correlated with each other ($r > 0.90$). On average, smaller values were obtained when fewer HBD-classes were considered, but the variance was then slightly larger (Supplementary Table S1). Interestingly F_{HBD-25}, restricted to recent HBD-segments, had the highest correlation with F_{PED} (0.83) among all measures considered in Supplementary Table S1. It was also highly correlated with F_{ROH} (0.99).

Correlations with homozygosity for different allele frequency (AF) classes and with whole genome homozygosity. Comparisons with homozygosity levels at alternate or private alleles grouped according to allele frequencies (Supplementary Figure S2AB), with marker homozygosity at different MAF categories (Supplementary Figure S2C) and with whole genome homozygosity (Supplementary Figure S2D) indicated that global measures F_{PED} and F_{ROH} including all pedigree generations or all ROH, performed the best. Nevertheless, measures based on the most recent generations of the pedigree (F_{PED-5G}) or recent ROH (> 5 Mb) had very similar correlations with the different homozygosity measures as F_{PED} and F_{ROH}, respectively, indicating that they captured most of the variation of these inbreeding coefficients. Estimators relying on the longest ROH only, F_{ROH-L}, had high correlations with homozygosity measures. These were lower than those achieved with F_{ROH}, but still higher than correlations observed with F_{PED}. Correlations continued to decrease with F_{ROH-M} and F_{ROH-S}. The values were still positive but below the levels obtained with F_{PED}. Finally, $F_{PED-OLD}$ presented almost null correlations with the different homozygosity measures.

With HBD-based measures, F_{HBD-25} and $F_{HBD-125}$ presented almost identical results (Supplementary Figure S4). When $F_{HBD-525}$ was computed using all identified HBD segments, correlations with homozygosity at alternate alleles for different AF classes (Supplementary Figure S4A) were higher for AF < 0.5, compared to HBD measures relying only on the longest HBD segments (F_{HBD-25}). With private alleles, $F_{HBD-525}$ had higher correlations with homozygosity at low frequency alleles (AF < 0.15; Supplementary Figure S4B). Interestingly, the correlation curves obtained with F_{HBD-25} were almost identical to those obtained with F_{ROH} and F_{HOM} (Supplementary Figure S4). As a result, F_{HBD-25} had one of the highest correlations with whole genome homozygosity (Supplementary Figure S4D).

Correlations with homozygous mutation load (HML). Similar trends were observed when estimators were compared to HML (Supplementary Figure S3). In general, F_{ROH} and F_{PED} had higher correlations with HML than measures using subsets of ROH or of pedigree-generations. Almost identical correlations, or slightly higher values, were achieved with F_{ROH-5} and F_{PED-5G}. F_{ROH-L} had consistently lower correlations with HML, although close to values observed with F_{ROH-5}. With F_{ROH-M}, correlations decreased further whereas with F_{ROH-S}, the values dropped more significantly. $F_{PED-OLD}$ had almost null correlations with different HML measures.

When using HBD-based measures, estimators based on HBD segments always had larger correlations with HML when all identified segments, including the shortest ones, were considered (Supplementary Figure S5). As observed previously, F_{HBD-25} presented very similar results to those observed with F_{ROH}.

Summary. Overall, the best results were obtained with $F_{HBD-525}$, F_{ROH} and F_{PED}, measures using all information. Nevertheless, F_{ROH-5} and F_{PED-5G} presented comparable correlations with homozygosity or HML, indicating that these measures of recent inbreeding captured most of the variation in F_{ROH} and F_{PED}, respectively. Differences between F_{HBD-25} and $F_{HBD-525}$ were more pronounced.

Supplementary Text S2

Comparison of inbreeding coefficients relying on maximum likelihood approaches

The F_{ML} measure from our study is the maximum likelihood estimator from Wang (2007), using genotypes of triad of individuals to estimate the nine condensed IBD states (Jacquard 1974). This method is implemented in COANCESTRY (Wang 2011) and the related R package (Pew et al. 2015). In the manuscript, we also described the F_{HBD} measure based on identification of homozygous-by-descent (HBD) segments proposed in Druet and Gautier (2017).

Both methods rely on the probabilities to observe genotypes conditionally on *F* and Hardy-Weinberg proportions. In both cases, genotypes come from a mixture of two distributions (autozygous *vs* allozygous) and *F* is estimated as the value maximizing the likelihood.

Here, we want to illustrate that when SNPs are considered independent in F_{HBD} (i.e. when the probability of coancestry change between markers is 1.0 for very distant ancestors separated by many generations of recombination), the two methods provide very similar estimators of the inbreeding coefficient.

Therefore, we estimated F_{HBD} with RZooROH (Bertrand et al. 2019) using a hidden Markov model with one HBD and one non-HBD class and with a rate equal to 250,000. With such a high rate, the probability of ancestry change between successive markers is close to 1 (i.e., the markers are independent).

The correlation between F_{ML} and F_{HBD} estimated as described above was equal to 0.9999 and the estimated linear regression was $F_{HBD} = -0.0001 + 1.0013 \times F_{ML}$. Both results illustrate that these estimators are extremely similar.

Supplementary Text S3

Estimation of inbreeding coefficients with low marker density

Methods. All genomic inbreeding coefficients were re-estimated with fewer markers. The new set of markers contained 5,977 SNPs from the Illumina BovineLD BeadChip instead of the 37,675 SNPs used previously.

F_{UNI}, F_{GRM}, F_{ML}, F_{HOM}, F_{ROH} and F_{HBD} were estimated using the same approach as before. For F_{ROH}, PLINK (Purcell et al. 2007) was run with the default options and the following changes: a minimum of 25 SNPs per ROH, at least 1 SNP per 500 Kb, a scanning window of 25 SNPs, a total length > 2 Mb and no heterozygous SNPs. Compared with options selected at higher density, the number of SNPs per ROH was reduced by 50%. This increased the risk of identifying false positive tracks (non-IBD). However, almost no ROH were detected when the number of SNPs was maintained at 50 per ROH, indicating that ROH are not recommended at this marker density.

Results. Results are reported in Supplementary Figures S19 to S21. Overall, although correlations were lower compared to estimators obtained with 37,675 SNPs, correlations remained high, in particular for the whole genome homozygosity (Supplementary Figure S19). The ranking of the methods and their behaviour was also in line with results obtained with more markers. The most striking difference was that F_{ROH} presented lower correlations, closer to values obtained with F_{PED} (see for instance Supplementary Figure S19). For estimation of locus-specific inbreeding coefficients (Supplementary Figure S21), F_{HBD} appeared more robust (i.e. smaller differences with results obtained with the 37,675 SNPs) as it uses information from succession of SNPs in a probabilistic framework. Consequently, it was the most efficient to capture regional scores, for both regional homozygosity and regional HML.

Supplementary Text S4

Comparison of inbreeding coefficients predicted in offspring with genotypes from parents

Methods. When mating two individuals, it is of interest to predict the inbreeding coefficient of their offspring. Here, we compared the predicted inbreeding coefficient based on 50K density genotypes from the 145 sequenced parents with the observed inbreeding levels in the 100 sequenced offspring. The inbreeding coefficient of an individual is also equal to the coancestry coefficient between its parents (Malécot 1948). Therefore, predicting the inbreeding coefficients in the offspring amounts to estimating the relatedness between their parents. For each inbreeding estimator previously used, we tried to use a related relationship measure. The pedigree additive relationship, the genomic relationships estimated as in Yang et al. (2011) and with the first method from VanRaden (2008) and the triadic likelihood approach (Wang 2007) were used to predict F_{PED}, F_{UNI}, F_{GRM} and F_{ML}, respectively. The genomic relationship presented by Yang et al. (2011) is a correlation between parental allelic dosages (Speed and Balding 2015) averaged over all SNPs, and therefore linked to F_{UNI}. Predicting F_{UNI} at one marker as the average F_{UNI} of the four possible combinations of gametes (i.e., four possible genotypes in the offspring) is indeed equivalent to estimate F_{GRM} using the genomic additive relationship from Yang et al. (2011) between the parents. To predict F_{HOM}, we relied on relatedness measures estimated from proportions of alleles that are identical-by-state (IBS) between two individuals. For instance, Eding and Meuwissen (2001) proposed to estimate relatedness based on a similarity index, whereas Weir and Goudet (2017) presented more recently a relatedness estimator that also relies on IBS proportions. The correlations between inbreeding coefficients estimated by these methods and F_{HOM} are equal to one (all these estimators rely on the observed homozygosity). Finally, we also used a method relying on identification of IBD segments shared between pairs of parents to predict F_{HBD}. We used a simplified approach relying on identification of ROH between the homologs from distinct parents (e.g., Pryce et al. 2012; de Cara et al. 2013; Luan et al. 2014). To that end, the four haplotypes from the parents were first obtained with Beagle 4.0 with the *ped* option (Browning and Browning 2007). To estimate the IBD probabilities at a locus between homologs from distinct parents (four possible pairs of paternal/maternal homologs), we applied the four HBD classes model to four virtual individuals created by combining two parental haplotypes (one from each parent). The additive relationship between the parents was finally computed as the average IBD probabilities (over the four possible combinations). As described in Supplementary Text S1, we considered HBD-measures relying on all identified HBD segments ($F_{HBD\text{-}525}$) or on the longest HBD segments associated with recent ancestors ($F_{HBD\text{-}25}$).

These inbreeding coefficients predicted using genotypes from the parents (with the 37,675 SNPs) were then compared to scores computed with the whole genome sequence data from the corresponding offspring.

Results. As expected, correlations with the different scores were lower when inbreeding coefficients were predicted from the genetic relationship between the parents, rather than estimated using the genotypes from the individual. Estimators giving more weight to rare alleles better predicted the homozygosity at rare alleles and HML, whereas the other measures were better predictors of the whole genome homozygosity (Supplementary Figures S20-22). The latter group of methods performed poorly to predict homozygosity at rare alleles in general except for HML at private missense variants (tolerated and deleterious) for which correlations were somehow larger (Supplementary Figure S21), close to the levels achieved by the first group of methods. Homozygosity at deleterious alleles was better predicted using all HBD segments ($F_{HBD\text{-}525}$) rather than long HBD segments only ($F_{HBD\text{-}25}$).

References

Bertrand AR, Kadri NK, Flori L, Gautier M, Druet T (2019) RZooRoH: An R package to characterize individual genomic autozygosity and identify homozygous-by-descent segments. *Methods Ecol Evol* **10**: 860–866.

Browning SR, Browning BL (2007) Rapid and accurate haplotype phasing and missing-data inference for whole-genome association studies by use of localized haplotype clustering. *Am J Hum Genet* **81**: 1084–1097.

de Cara MÁR, Villanueva B, Toro MÁ, Fernández J (2013) Using genomic tools to maintain diversity and fitness in conservation programmes. *Mol Ecol* **22**: 6091–6099.

Druet T, Gautier M (2017) A model-based approach to characterize individual inbreeding at both global and local genomic scales. *Mol Ecol* **26**: 5820–5841.

Eding H, Meuwissen THE (2001) Marker-based estimates of between and within population kinships for the conservation of genetic diversity. *J Anim Breed Genet* **118**: 141–159.

Jacquard A (1974) *The Genetic Structure of Populations*. Springer-Verlag: New-York.

Luan T, Yu X, Dolezal M, Bagnato A, Meuwissen TH (2014) Genomic prediction based on runs of homozygosity. *Genet Sel Evol* **46**: 64.

Malécot G (1948) Mathématiques de l'hérédité. Masson et Cie, Paris.

Pew J, Muir PH, Wang J, Frasier TR (2015) related: an R package for analysing pairwise relatedness from codominant molecular markers. *Mol Ecol Resour* **15**: 557–561.

Pryce JE, Hayes BJ, Goddard ME (2012) Novel strategies to minimize progeny inbreeding while maximizing genetic gain using genomic information. *J Dairy Sci* **95**: 377–388.

Purcell S, Neale B, Todd-Brown K, Thomas L, Ferreira MAR, Bender D, et al. (2007) PLINK: a tool set for whole-genome association and population-based linkage analyses. *Am J Hum Genet* **81**: 559–575.

Solé M, Gori A-S, Faux P, Bertrand A, Farnir F, Gautier M, et al. (2017) Age-based partitioning of individual genomic inbreeding levels in Belgian Blue cattle. *Genet Sel Evol* **49**: 92.

Speed D, Balding DJ (2015) Relatedness in the post-genomic era: is it still useful? *Nat Rev Genet* **16**: 33.

VanRaden PM (2008) Efficient methods to compute genomic predictions. *J Dairy Sci* **91**: 4414–4423.

Wang J (2007) Triadic IBD coefficients and applications to estimating pairwise relatedness. *Genet Res* **89**: 135–153.

Wang J (2011) COANCESTRY: a program for simulating, estimating and analysing relatedness and inbreeding coefficients. *Mol Ecol Resour* **11**: 141–145.

Weir BS, Goudet J (2017) A unified characterization of population structure and relatedness. *Genetics* **206**: 2085–2103.

Yang J, Lee SH, Goddard ME, Visscher PM (2011) GCTA: a tool for genome-wide complex trait analysis. *Am J Hum Genet* **88**: 76–82.

Supplementary Table S1. Summary statistics for different inbreeding coefficients estimated for the 145 sequenced parents using a 50K marker panel.

Estimators	mean F	Variance F	min F	max F
F_{UNI}	-0.020	0.00125	-0.106	0.147
F_{GRM}	-0.022	0.00337	-0.204	0.218
F_{ML}	0.006	0.00028	0.000	0.119
F_{HOM}	-0.022	0.00144	-0.098	0.111
F_{HBD-25}	0.076	0.00120	0.011	0.201
$F_{HBD-125}$	0.101	0.00119	0.045	0.219
$F_{HBD-525}$	0.111	0.00103	0.049	0.219
F_{PED}	0.046	0.00079	0.007	0.164
F_{PED-5G}	0.033	0.00079	0.000	0.162
$F_{PED-OLD}$	0.013	0.00001	0.001	0.031
F_{ROH}	0.066	0.00101	0.010	0.183
F_{ROH-S}	0.011	0.00003	0.001	0.029
F_{ROH-M}	0.020	0.00009	0.003	0.048
F_{ROH-L}	0.035	0.00065	0.000	0.143

F_{UNI} = inbreeding coefficient based on the correlation between uniting gametes
F_{GRM} = inbreeding coefficient estimated with the first method proposed by VanRaden (2008) and based on the diagonal elements of the genomic relationship matrix (dividing all SNP contributions by the same denominator)
F_{ML} = maximum likelihood estimator of the inbreeding coefficient
F_{HOM} = excess homozygosity estimator
F_{HBD-T} = inbreeding coefficient estimated as the probability of belonging to any of the HBD classes with a rate higher or equal to T averaged over the whole genome
F_{PED} = inbreeding coefficient estimated from pedigree data
F_{PED-5G} = inbreeding coefficient estimated from five generations of pedigree
$F_{PED-OLD}$ = inbreeding coefficient estimated from ancient ancestors from the pedigree, and equal to F_{PED} - F_{PED-5G}
F_{ROH} = inbreeding coefficient estimated from ROH longer than 2 Mb
F_{ROH-S} = inbreeding coefficient estimated from short ROH (2-5 Mb)
F_{ROH-M} = inbreeding coefficient estimated from intermediate ROH (5-10 Mb)
F_{ROH-L} = inbreeding coefficient estimated from long ROH (> 10 Mb)

Supplementary Table S2. Correlation coefficients between individual inbreeding coefficients estimated with different methods for the 145 sequenced parents and using the 50K panel.

	F_{GRM}	F_{ML}	F_{HOM}	F_{HBD-25}	$F_{HBD-125}$	$F_{HBD-525}$	F_{PED}	F_{PED-5G}	$F_{PED-OLD}$	F_{ROH}	F_{ROH-S}	F_{ROH-M}	F_{ROH-L}
F_{UNI}	0.878	0.761	0.696	0.644	0.610	0.821	0.468	0.462	0.021	0.656	0.198	0.435	0.622
F_{GRM}		0.602	0.285	0.228	0.189	0.471	0.104	0.117	-0.049	0.244	0.024	0.152	0.244
F_{ML}			0.572	0.589	0.537	0.673	0.518	0.545	-0.103	0.610	0.172	0.308	0.615
F_{HOM}				0.960	0.962	0.963	0.766	0.731	0.131	0.959	0.389	0.629	0.892
F_{HBD-25}					0.976	0.950	0.832	0.795	0.139	0.989	0.411	0.676	0.908
$F_{HBD-125}$						0.940	0.809	0.763	0.170	0.971	0.389	0.654	0.898
$F_{HBD-525}$							0.759	0.728	0.114	0.952	0.349	0.629	0.892
F_{PED}								0.966	0.126	0.821	0.300	0.596	0.749
F_{PED-5G}									-0.136	0.790	0.256	0.544	0.738
$F_{PED-OLD}$										0.113	0.165	0.195	0.038
F_{ROH}											0.347	0.658	0.941
F_{ROH-S}												0.276	0.137
F_{ROH-M}													0.405

F_{UNI} = inbreeding coefficient based on the correlation between uniting gametes; F_{GRM} = inbreeding coefficient estimated with the first method proposed by VanRaden (2008) and based on the diagonal elements of the genomic relationship matrix (dividing all SNP contributions by the same denominator); F_{ML} = maximum likelihood estimator of the inbreeding coefficient; F_{HOM} = excess homozygosity estimator; F_{HBD-T} = inbreeding coefficient estimated as the probability to belong to any of the HBD classes with a rate ≤ T, averaged over the whole genome; F_{HOM} = inbreeding coefficient based on the proportion of homozygous SNPs; F_{PED} = inbreeding coefficient estimated from pedigree data; F_{PED-5G} = inbreeding coefficient estimated from five generations of pedigree; $F_{PED-OLD}$ = inbreeding coefficient estimated from ancient ancestors from the pedigree, and equal to F_{PED} - F_{PED-5G} ; F_{ROH} = inbreeding coefficient estimated from ROH longer than 2 Mb; F_{ROH-S} = inbreeding coefficient estimated from short ROH (2-5 Mb); F_{ROH-M} = inbreeding coefficient estimated from intermediate ROH (5-10 Mb); F_{ROH-L} = inbreeding coefficient estimated from long ROH (> 10 Mb)

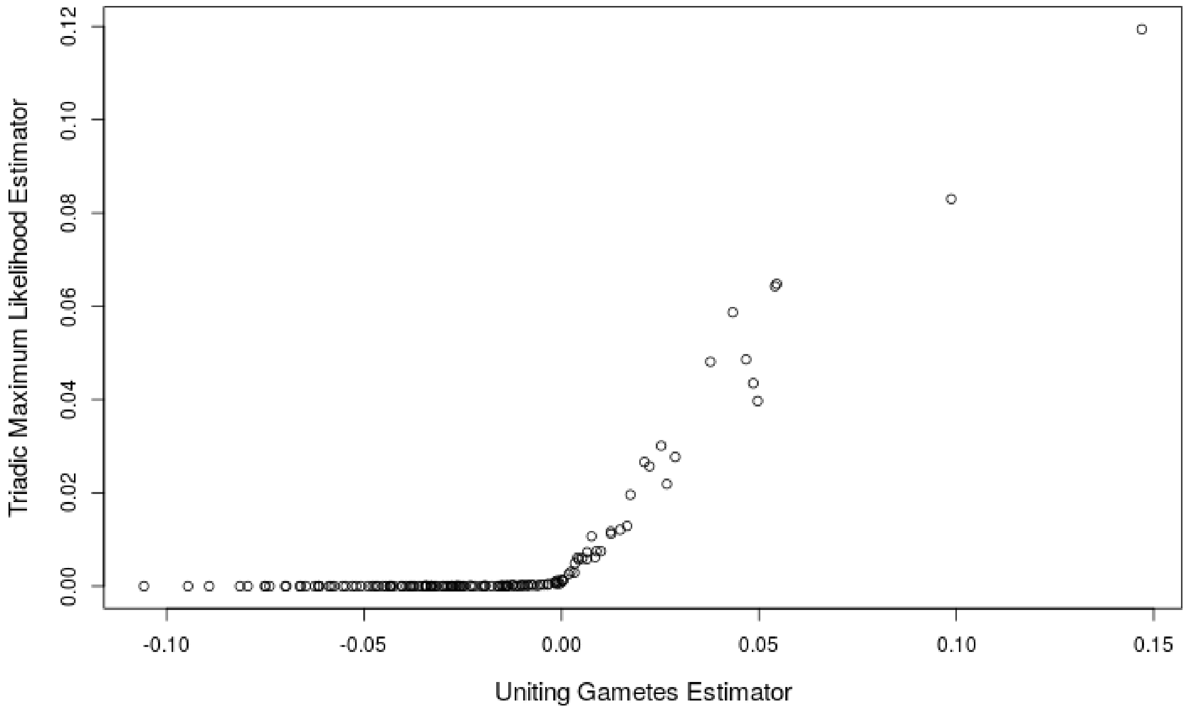

Supplementary Figure S1. Inbreeding coefficients measured in the parents with the uniting gametes method and with the triadic maximum likelihood estimator.

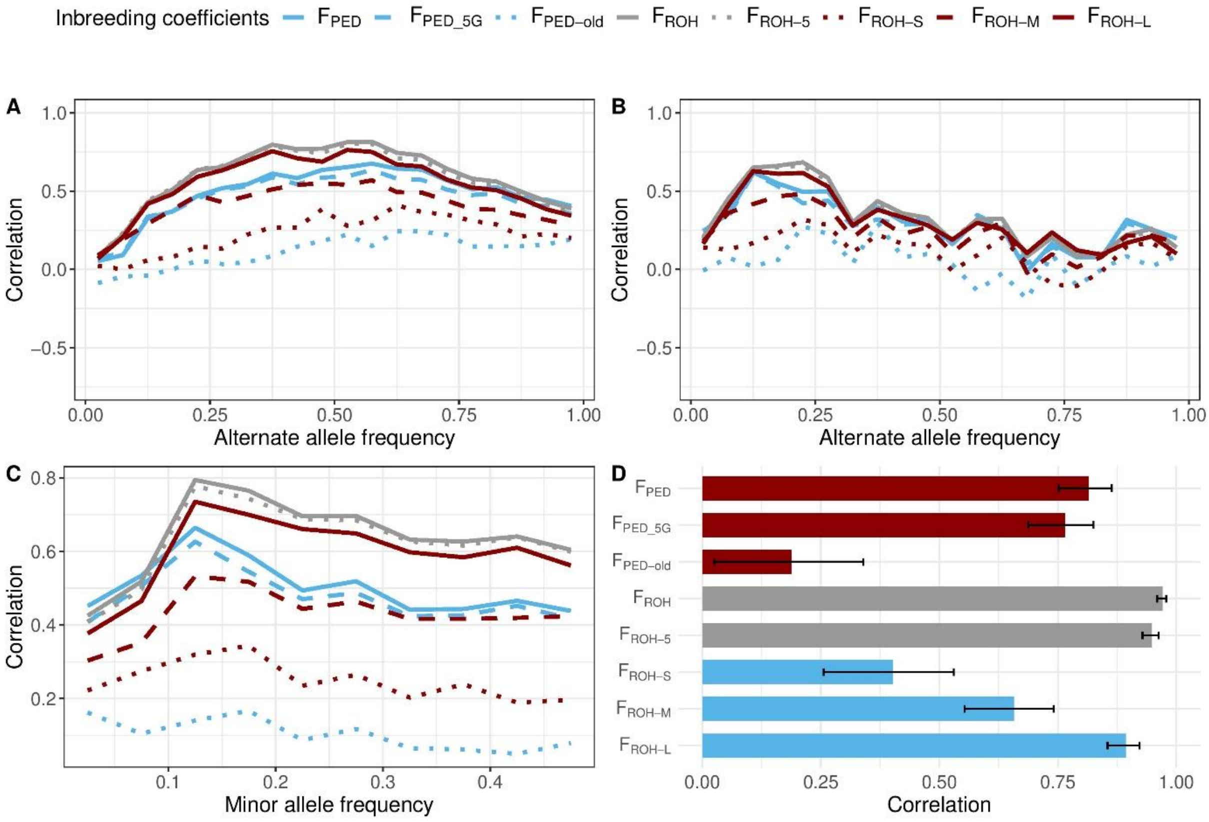

Supplementary Figure S2. Correlation coefficients between individual levels of recent and ancient inbreeding estimated with 37,675 SNPs and scores obtained from the whole-genome sequence data in 145 individuals. A. Correlation with homozygosity at alternate alleles grouped according to their allele frequency. B. Correlation with homozygosity at private alleles (young alleles) grouped according to their allele frequency. C. Correlation with global marker homozygosity (counted at both reference and alternate alleles) as a function of minor allele frequency. D. Correlation with whole-genome homozygosity. The error bars represent the 95% confidence intervals.

The inbreeding measures related to recent or ancient inbreeding are: $F_{PED\text{-}5G}$, the inbreeding coefficient estimated from five generations of pedigree; $F_{PED\text{-}OLD}$, the inbreeding coefficient estimated from ancient ancestors from the pedigree, and equal to F_{PED} - $F_{PED\text{-}5G}$; F_{ROH}, the inbreeding coefficient estimated from ROH longer than 2 Mb; $F_{ROH\text{-}S}$, the inbreeding coefficient estimated from short ROH (2-5 Mb); $F_{ROH\text{-}M}$, the inbreeding coefficient estimated from intermediate ROH (5-10 Mb); $F_{ROH\text{-}L}$, the inbreeding coefficient estimated from long ROH (> 10 Mb); $F_{ROH\text{-}5}$, the inbreeding coefficient estimated from ROH longer than 5 Mb.

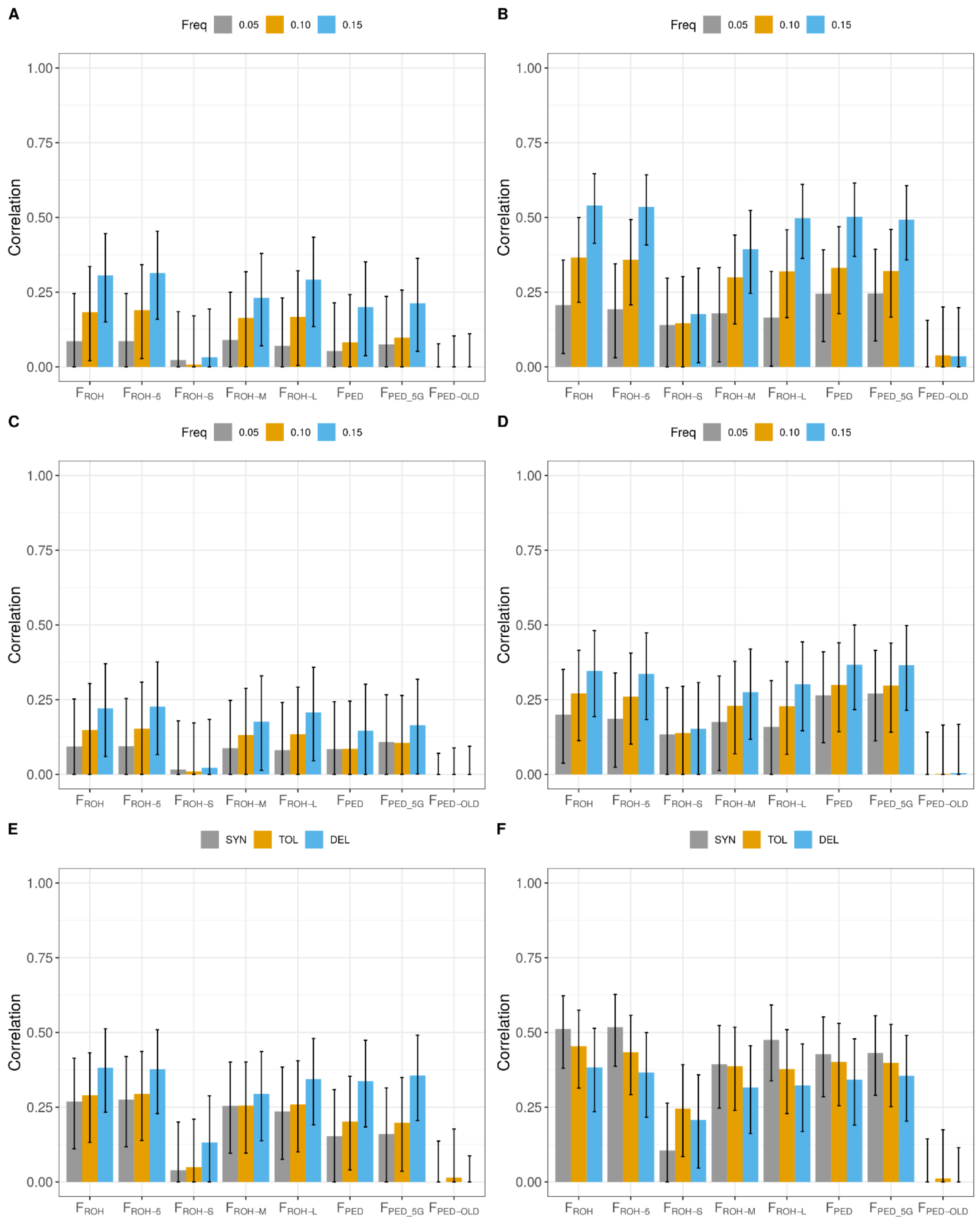

Supplementary Figure S3. Correlation coefficients between individual levels of recent and ancient inbreeding estimated with 37,675 SNPs and homozygous mutation load (HML) computed using alternate (A, C, E) and private alternate (B, D, F) alleles from the whole-genome sequence data in 145 individuals. A and B. Correlation with HML estimated with allele frequency thresholds of 0.05, 0.10 and 0.15. C and D. Correlation with weighted HML estimated with allele frequency thresholds of 0.05, 0.10 and 0.15. E and F. Correlation with HML estimated with synonymous (SYN), tolerated (TOL) and deleterious (DEL) missense variants and using an allele frequency threshold of 0.15. The error bars represent the 95% confidence intervals and are truncated at 0.

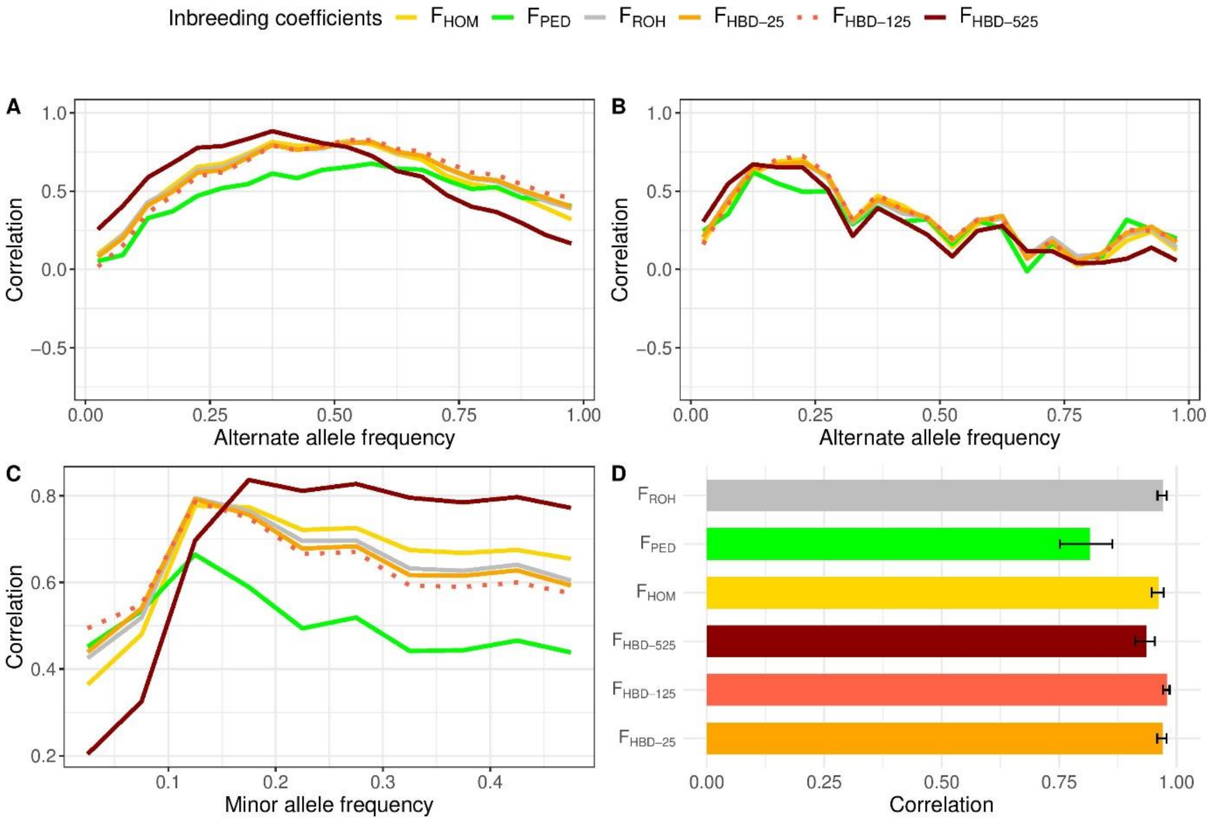

Supplementary Figure S4. Correlation coefficients between individual HBD-based inbreeding coefficients estimated with 37,675 SNPs and scores obtained from the whole-genome sequence data in 145 individuals. A. Correlation with homozygosity at alternate alleles grouped according to their allele frequency. B. Correlation with homozygosity at private alleles (young alleles) grouped according to their allele frequency. C. Correlation with global marker homozygosity (counted at both reference and alternate alleles) as a function of minor allele frequency. D. Correlation with whole-genome homozygosity. The error bars represent the 95% confidence intervals.

The HBD-based inbreeding coefficients $F_{HBD\text{-}T}$ were obtained by considering only HBD-classes with a rate $R_k \leq T$, setting the base population to approximately $0.5 \times T$ generations ago. Lower values of T correspond to more recent inbreeding and to consider only longer HBD segments. Here, we computed $F_{HBD\text{-}25}$, $F_{HBD\text{-}125}$ and $F_{HBD\text{-}525}$.

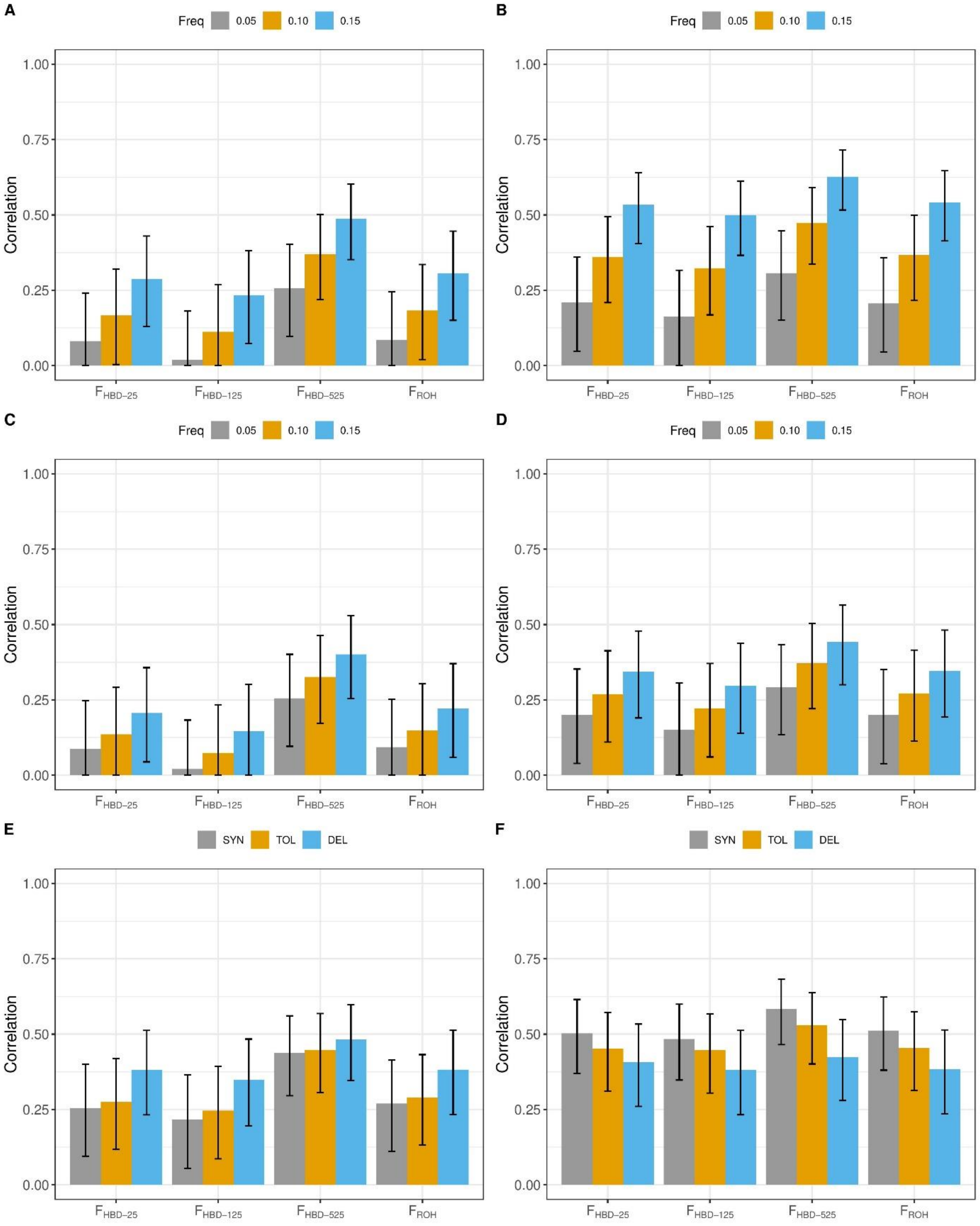

Supplementary Figure S5. Correlation coefficients between individual HBD-based inbreeding coefficients estimated with 37,675 SNPs and homozygous mutation load (HML) computed using alternate (A, C, E) and private alternate (B, D, F) alleles from the whole-genome sequence data in 145 individuals. A and B. Correlation with HML estimated with allele frequency thresholds of 0.05, 0.10 and 0.15. C and D. Correlation with weighted HML estimated with allele frequency thresholds of 0.05, 0.10 and 0.15. E and F. Correlation with HML estimated with synonymous (SYN), tolerated (TOL) and deleterious (DEL) missense variants and using an allele frequency threshold of 0.15. The error bars represent the 95% confidence intervals and are truncated at 0.

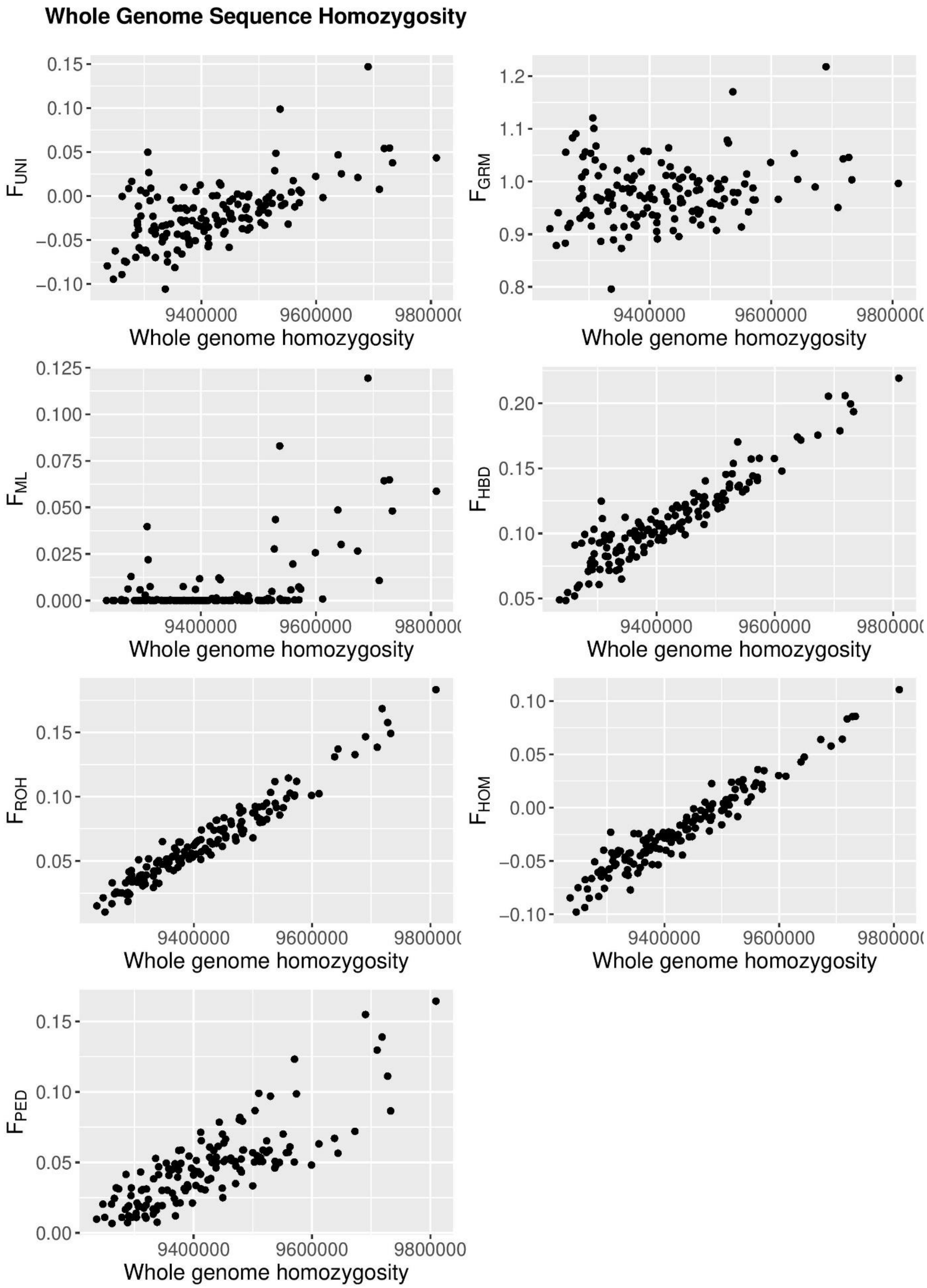

Supplementary Figure S6. Scatterplots between the whole genome homozygosity and the seven compared inbreeding coefficients, F_{UNI}, F_{GRM}, F_{ML}, F_{HBD}, F_{ROH}, F_{HOM} and F_{PED}.

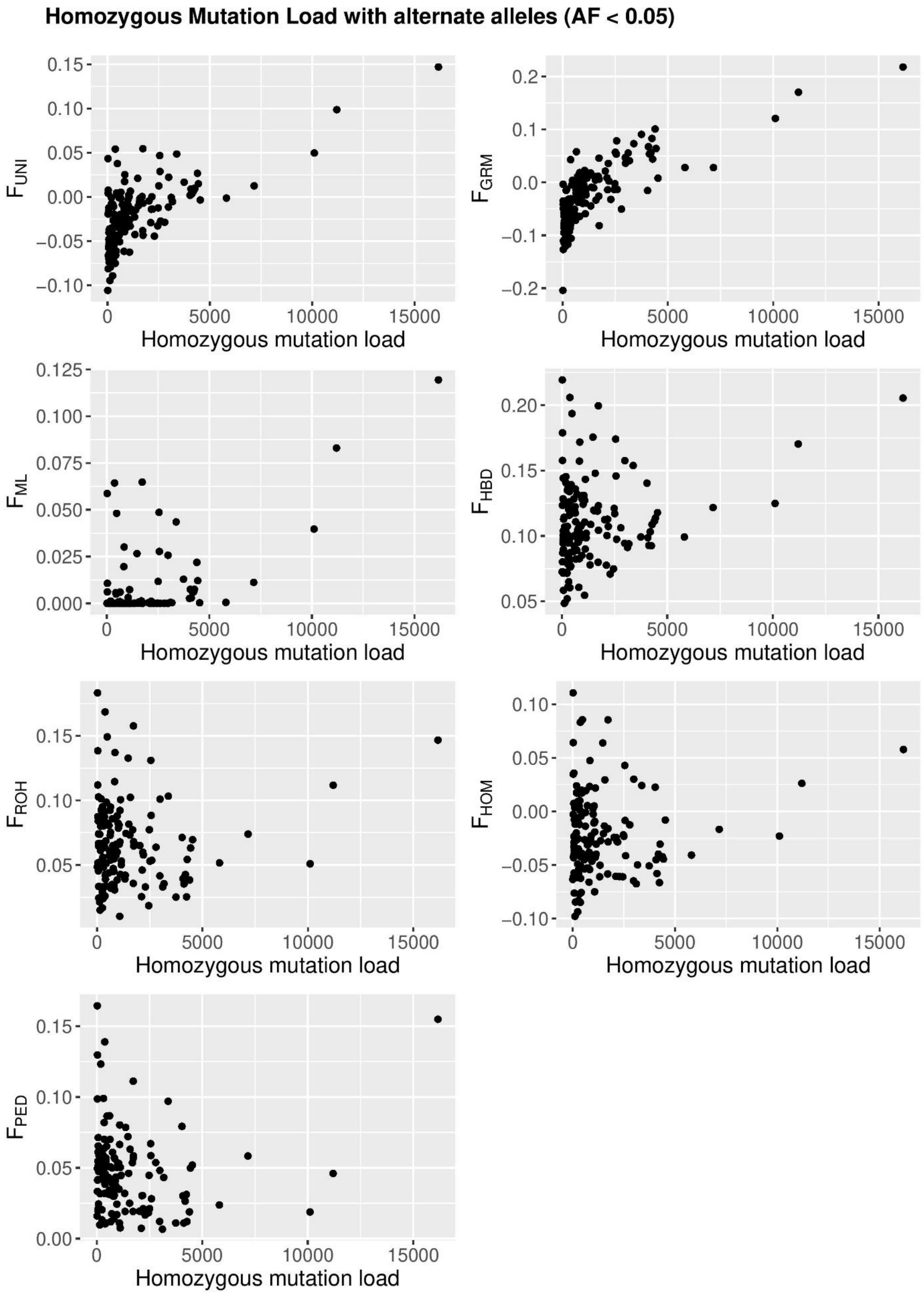

Supplementary Figure S7. Scatterplots between the homozygosity mutation load at alternate alleles with AF < 0.05 and the seven compared inbreeding coefficients, F_{UNI}, F_{GRM}, F_{ML}, F_{HBD}, F_{ROH}, F_{HOM} and F_{PED}.

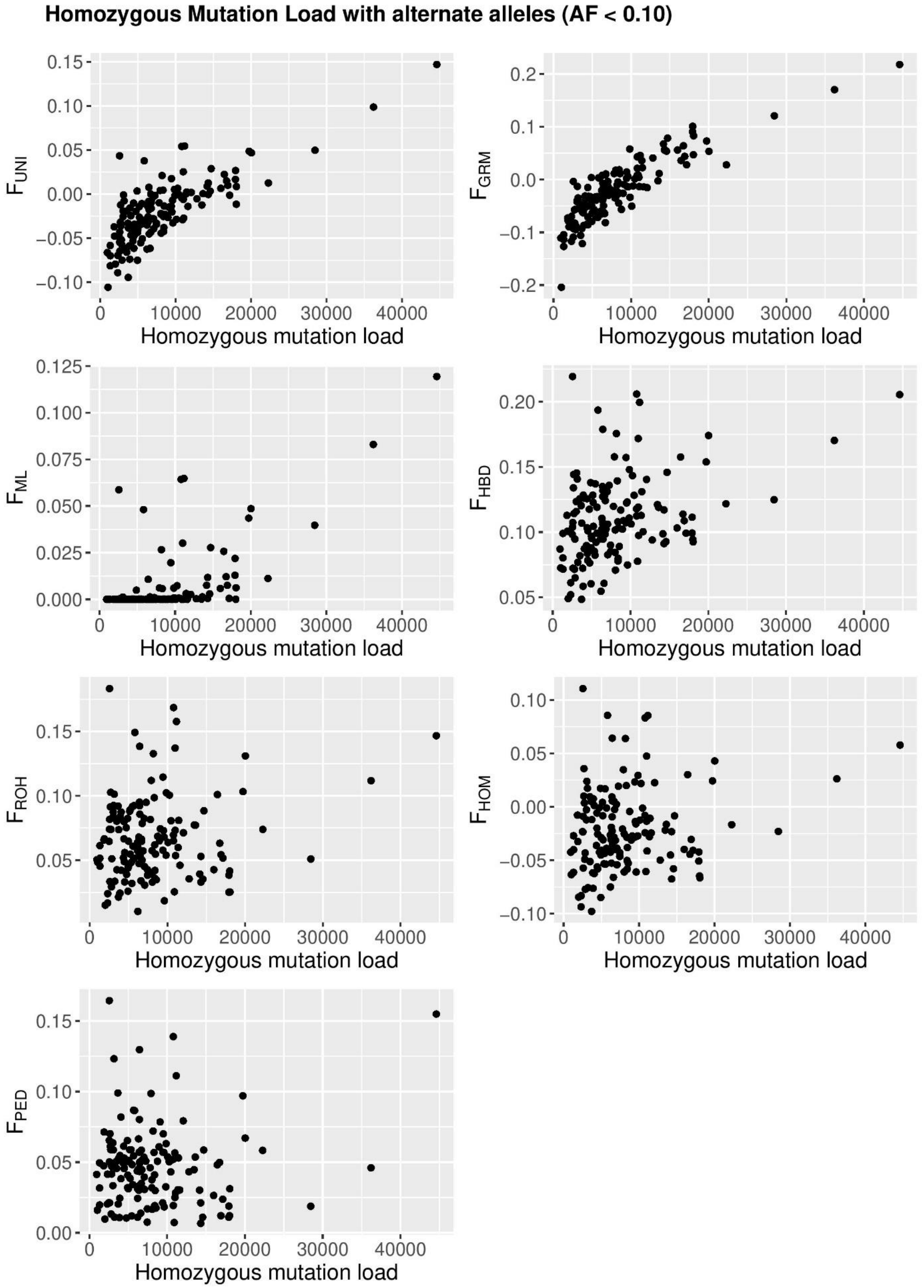

Supplementary Figure S8. Scatterplots between the homozygosity mutation load at alternate alleles with AF < 0.10 and the seven compared inbreeding coefficients, F_{UNI}, F_{GRM}, F_{ML}, F_{HBD}, F_{ROH}, F_{HOM} and F_{PED}.

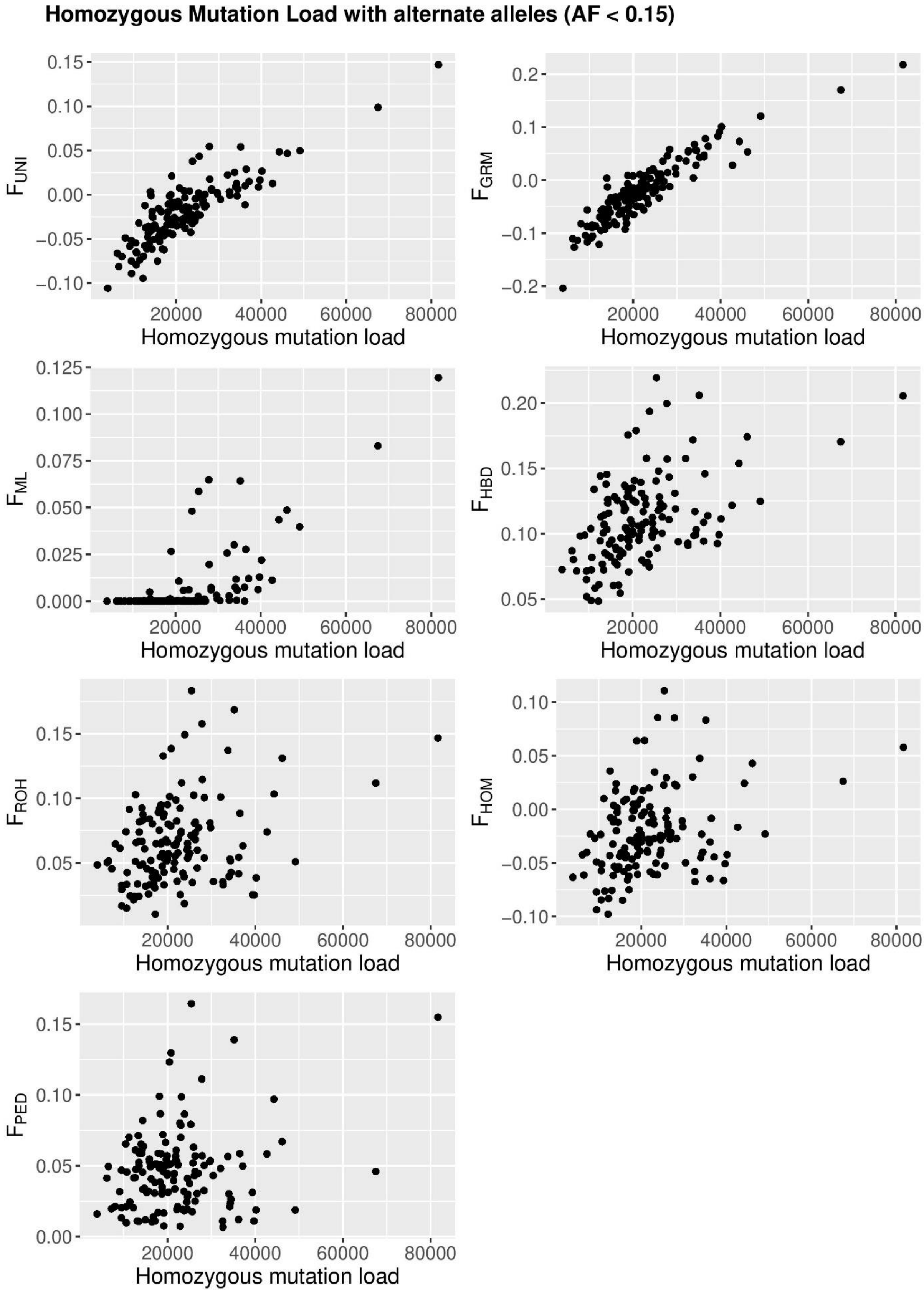

Supplementary Figure S9. Scatterplots between the homozygosity mutation load at alternate alleles with AF < 0.15 and the seven compared inbreeding coefficients, F_{UNI}, F_{GRM}, F_{ML}, F_{HBD}, F_{ROH}, F_{HOM} and F_{PED}.

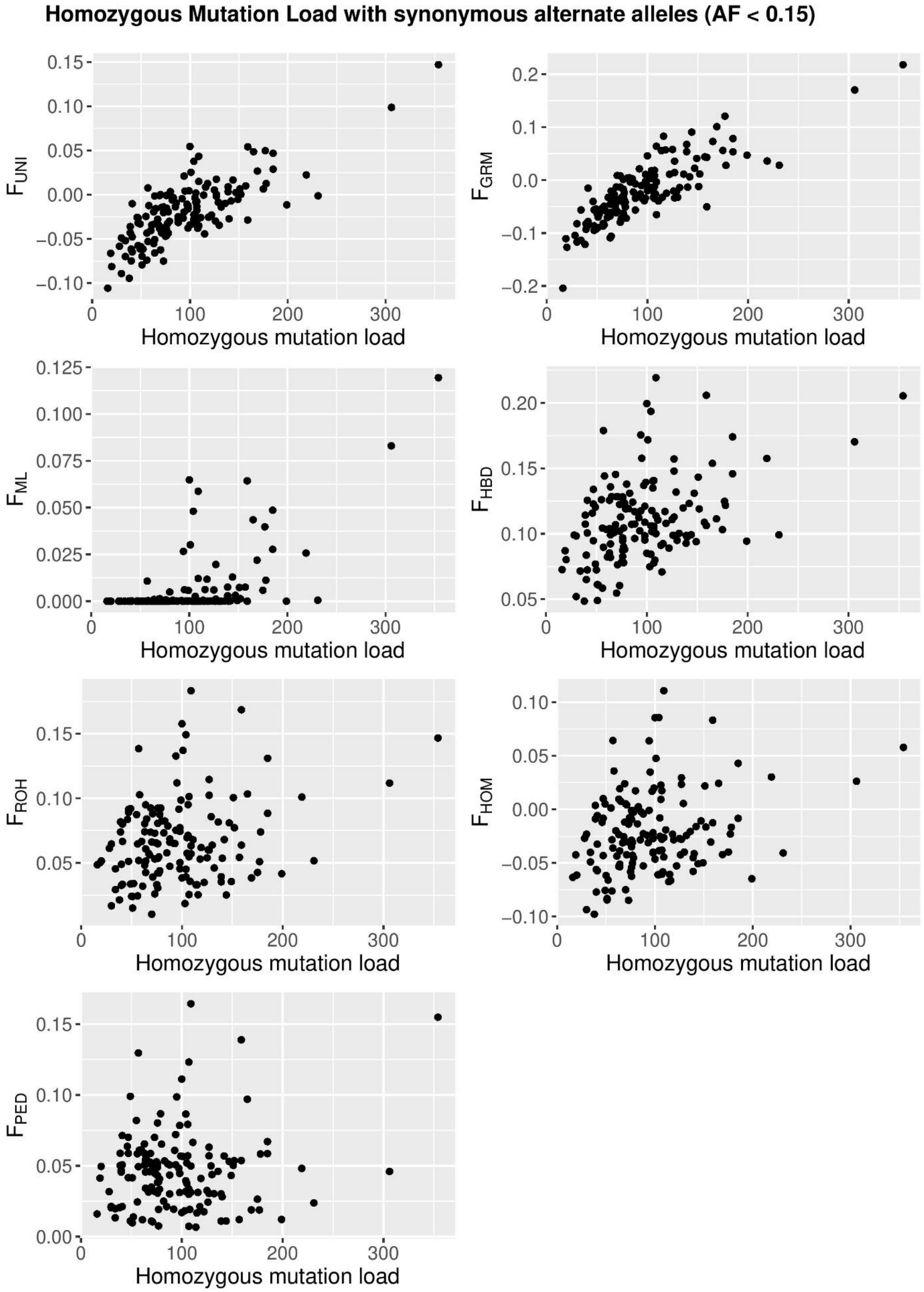

Supplementary Figure S10. Scatterplots between the homozygosity mutation load at synonymous alternate alleles with AF < 0.15 and the seven compared inbreeding coefficients, F_{UNI}, F_{GRM}, F_{ML}, F_{HBD}, F_{ROH}, F_{HOM} and F_{PED}.

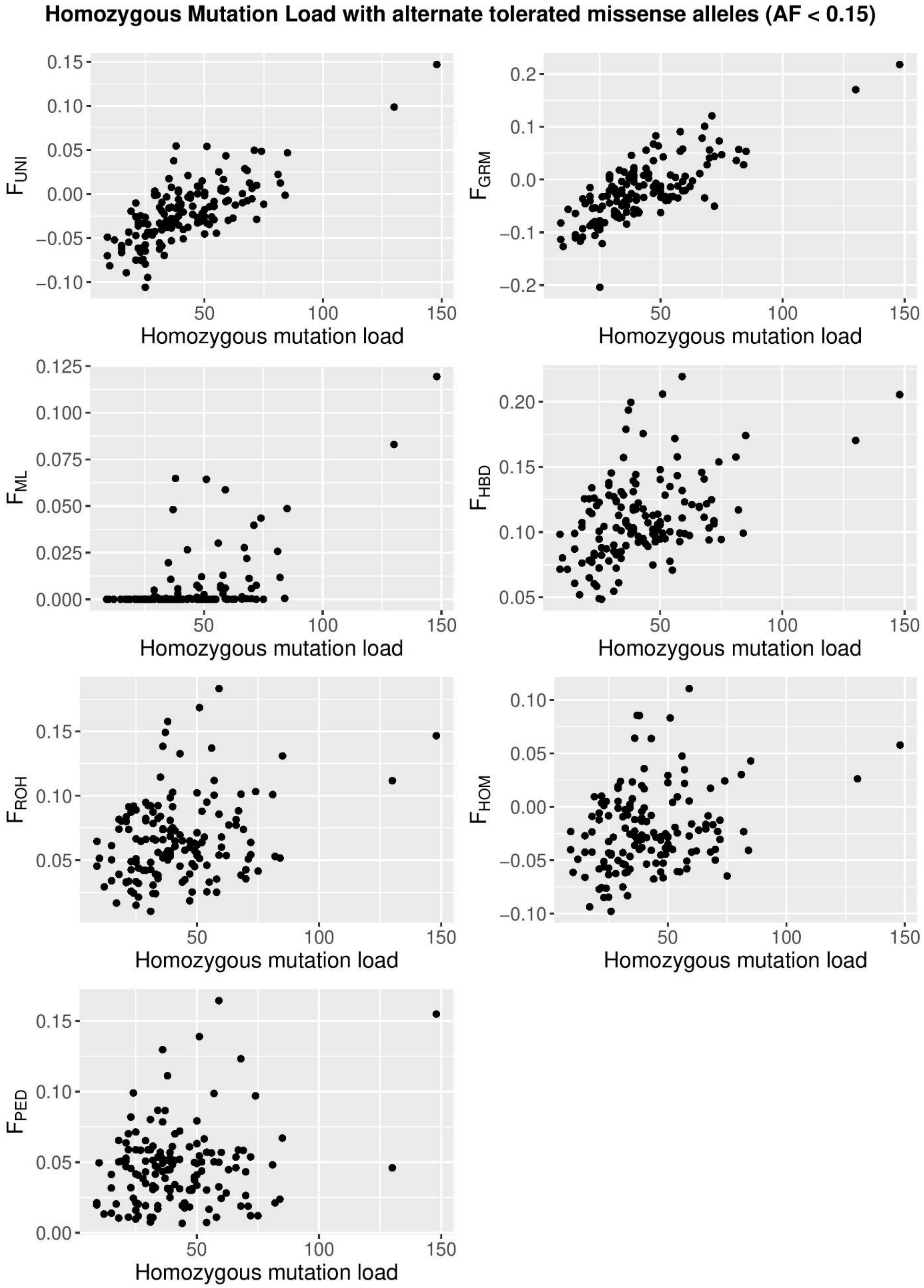

Supplementary Figure S11. Scatterplots between the homozygosity mutation load at tolerated missense alternate alleles with AF < 0.15 and the seven compared inbreeding coefficients, F_{UNI}, F_{GRM}, F_{ML}, F_{HBD}, F_{ROH}, F_{HOM} and F_{PED}.

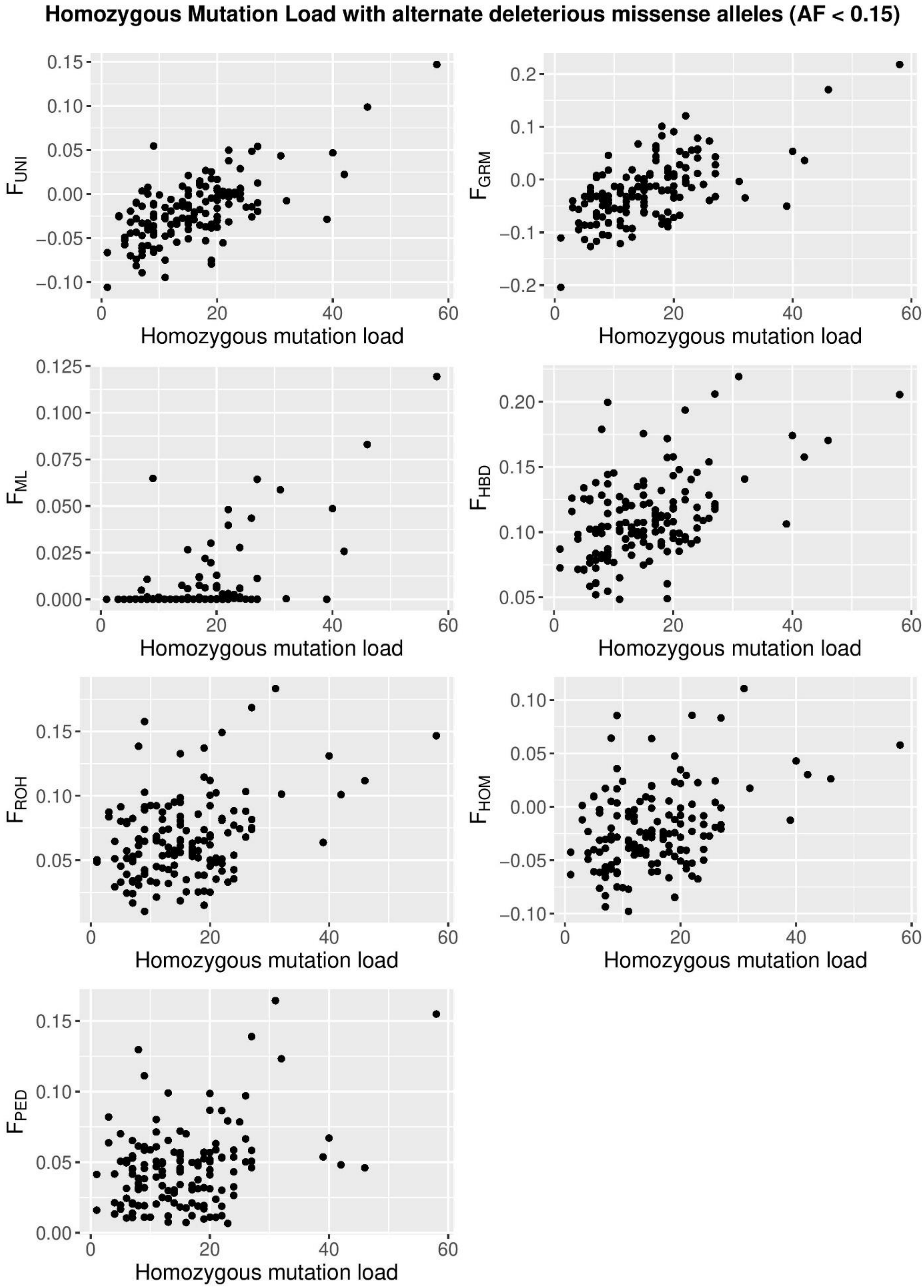

Supplementary Figure S12. Scatterplots between the homozygosity mutation load at deleterious missense alternate alleles with AF < 0.15 and the seven compared inbreeding coefficients, F_{UNI}, F_{GRM}, F_{ML}, F_{HBD}, F_{ROH}, F_{HOM} and F_{PED}.

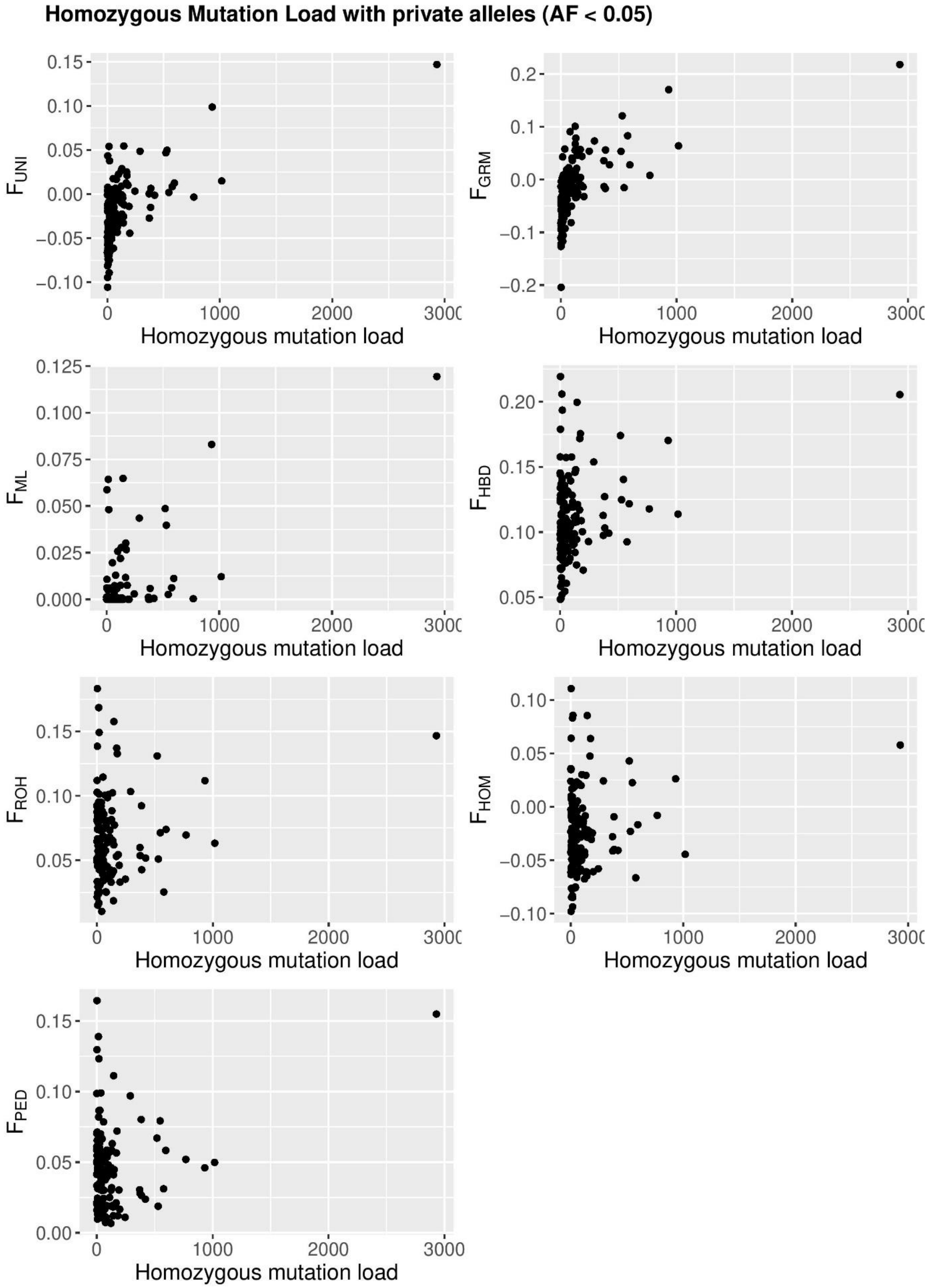

Supplementary Figure S13. Scatterplots between the homozygosity mutation load at alternate alleles with AF < 0.05 and the seven compared inbreeding coefficients, F_{UNI}, F_{GRM}, F_{ML}, F_{HBD}, F_{ROH}, F_{HOM} and F_{PED}.

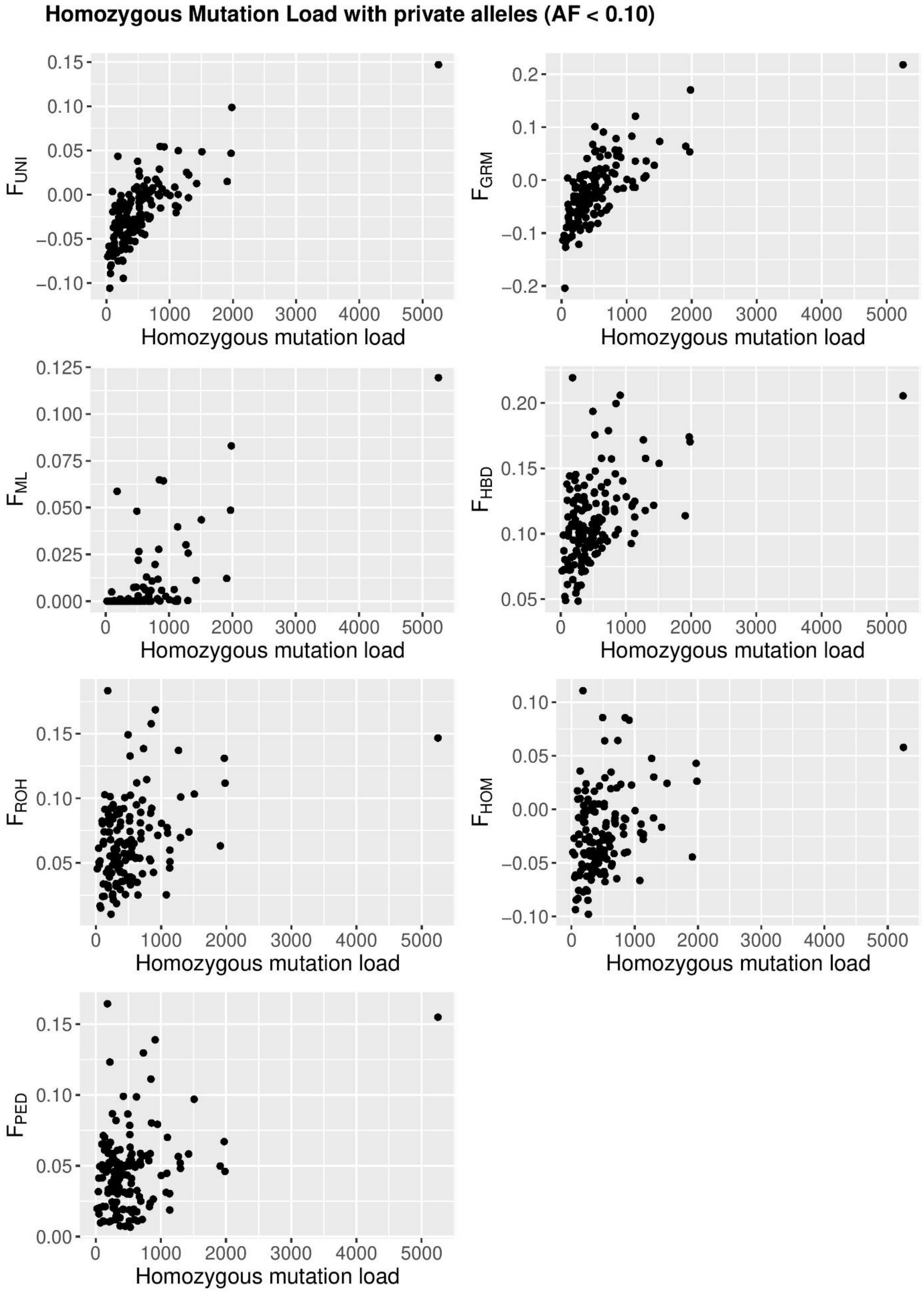

Supplementary Figure S14. Scatterplots between the homozygosity mutation load at private alleles with AF < 0.10 and the seven compared inbreeding coefficients, F_{UNI}, F_{GRM}, F_{ML}, F_{HBD}, F_{ROH}, F_{HOM} and F_{PED}.

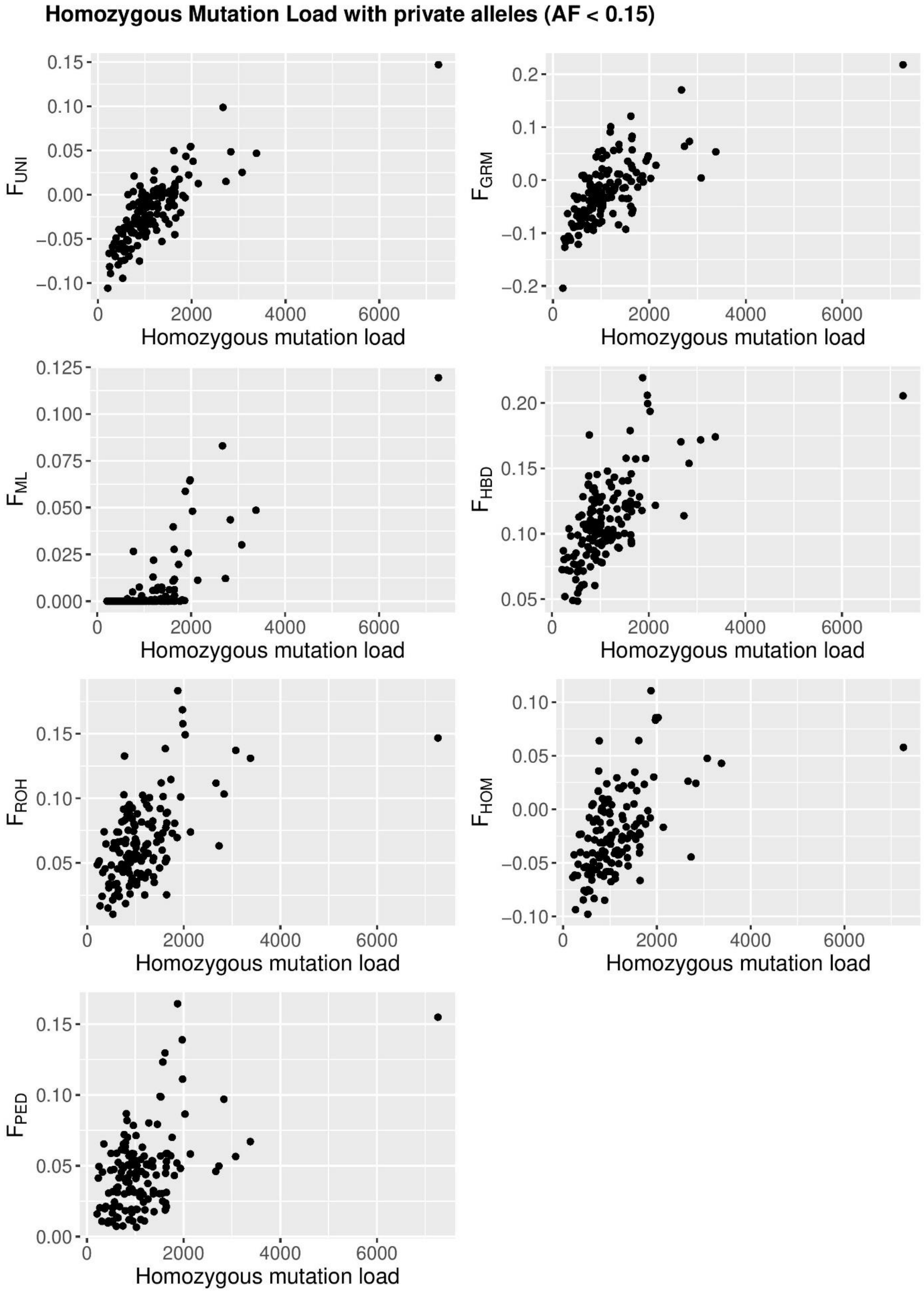

Supplementary Figure S15. Scatterplots between the homozygosity mutation load at private alleles with AF < 0.15 and the seven compared inbreeding coefficients, F_{UNI}, F_{GRM}, F_{ML}, F_{HBD}, F_{ROH}, F_{HOM} and F_{PED}.

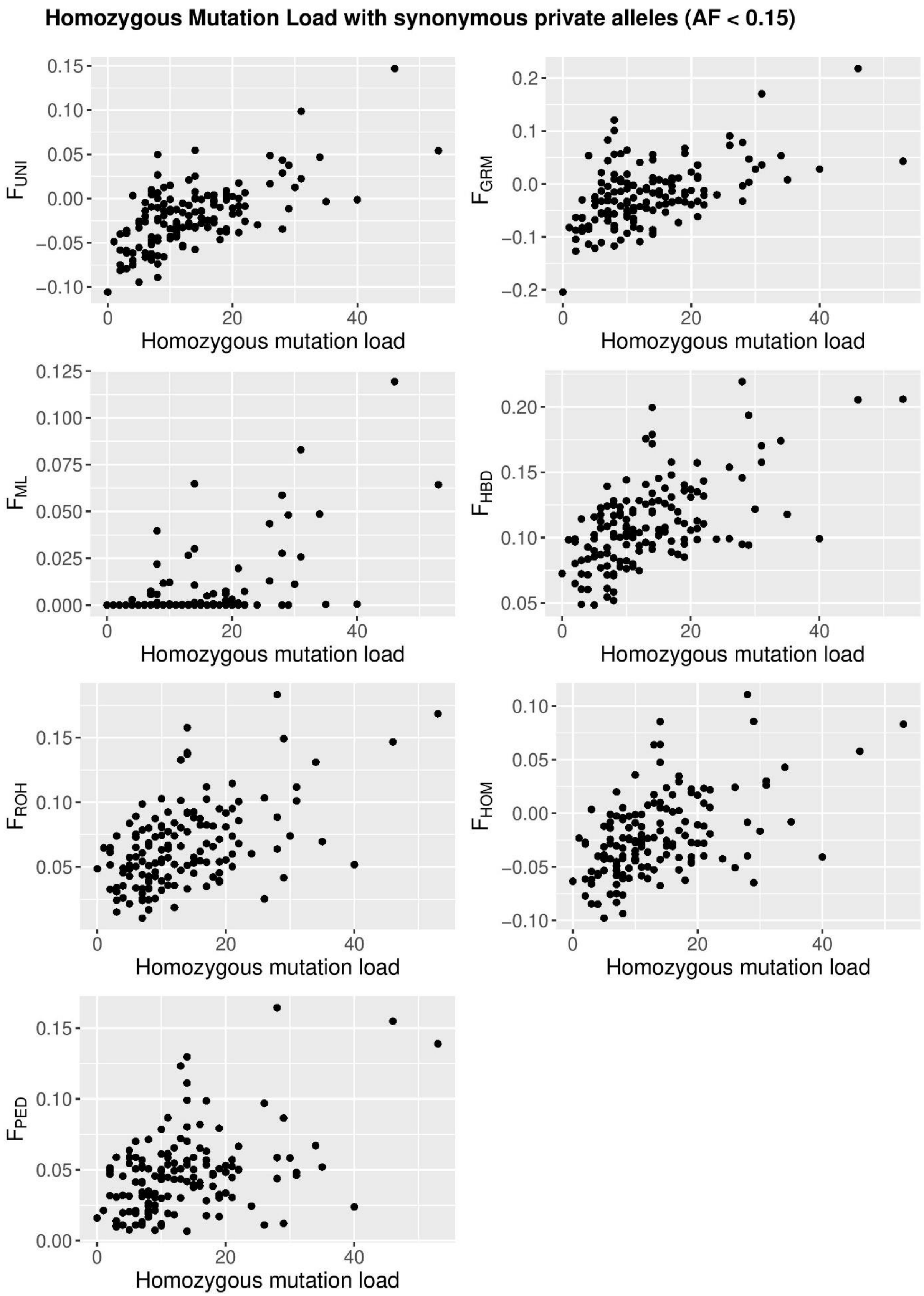

Supplementary Figure S16. Scatterplots between the homozygosity mutation load at synonymous private alleles with AF < 0.15 and the seven compared inbreeding coefficients, F_{UNI}, F_{GRM}, F_{ML}, F_{HBD}, F_{ROH}, F_{HOM} and F_{PED}.

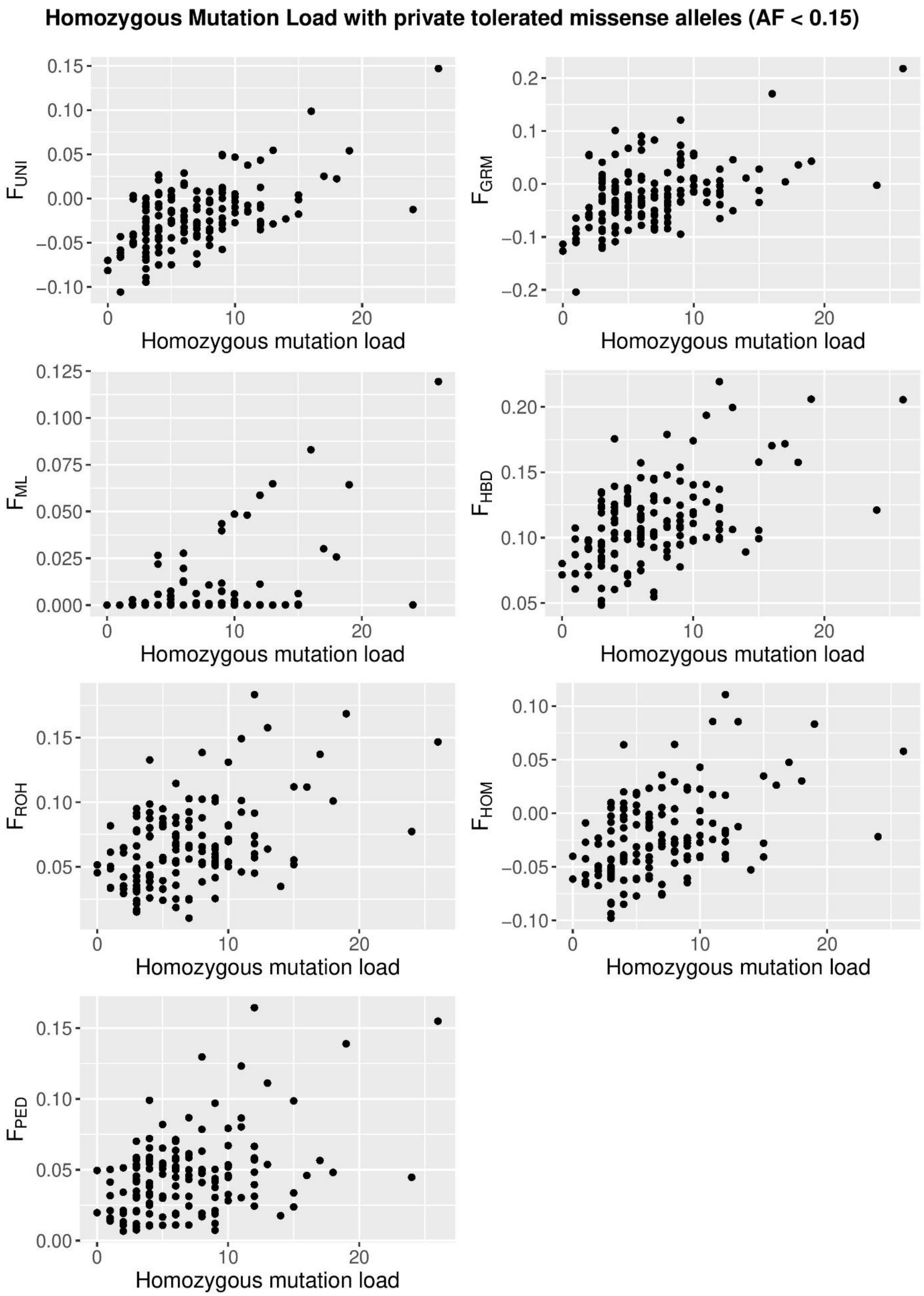

Supplementary Figure S17. Scatterplots between the homozygosity mutation load at tolerated missense private alleles with AF < 0.15 and the seven compared inbreeding coefficients, F_{UNI}, F_{GRM}, F_{ML}, F_{HBD}, F_{ROH}, F_{HOM} and F_{PED}.

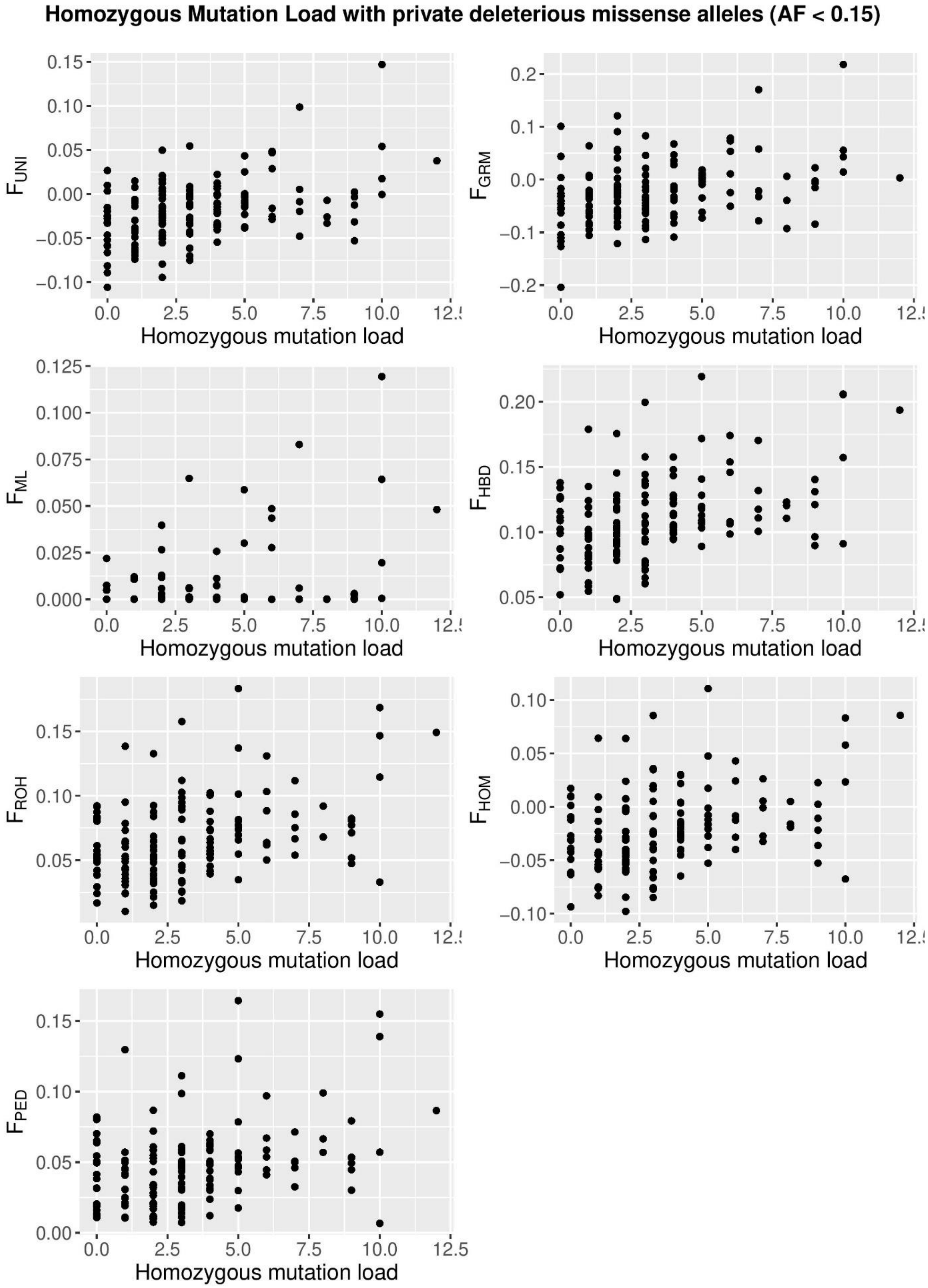

Supplementary Figure S18. Scatterplots between the homozygosity mutation load at deleterious missense private alleles with AF < 0.15 and the seven compared inbreeding coefficients, F_{UNI}, F_{GRM}, F_{ML}, F_{HBD}, F_{ROH}, F_{HOM} and F_{PED}.

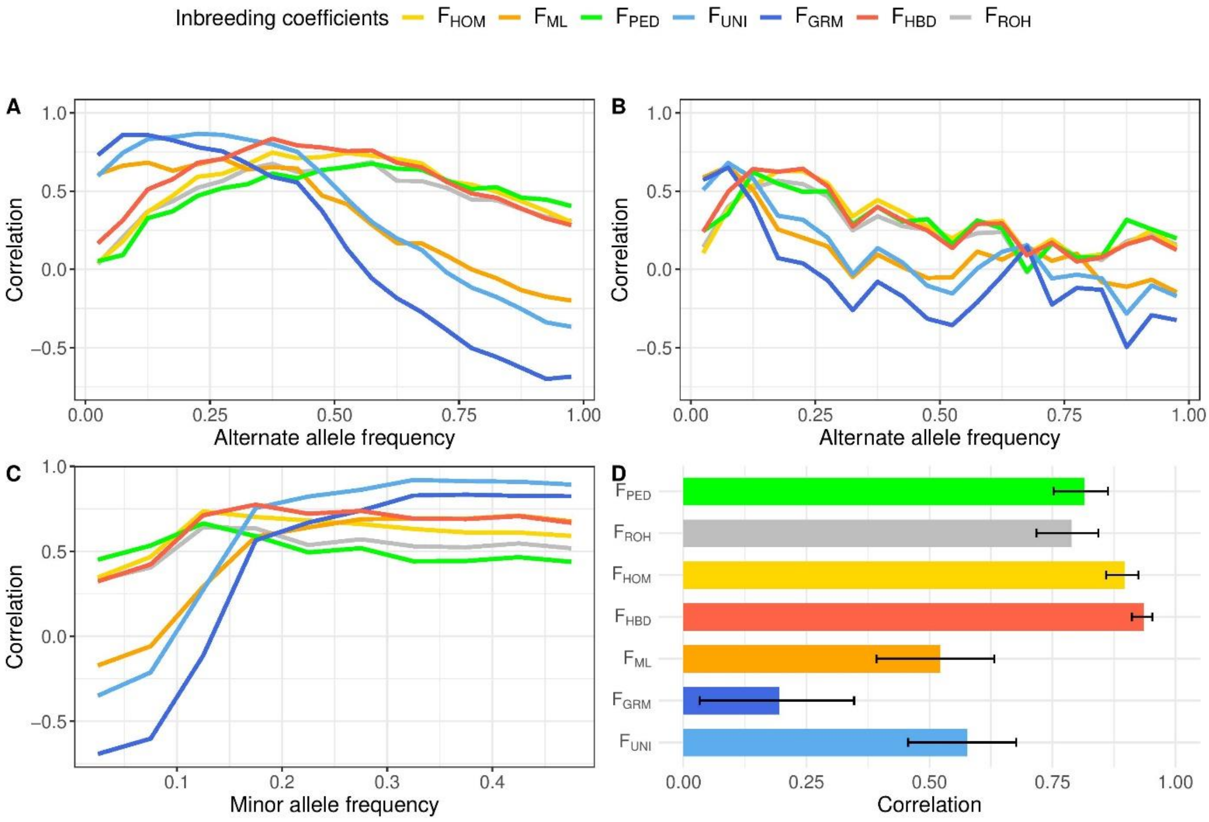

Supplementary Figure S19. Correlation coefficients between individual inbreeding measures estimated at lower marker density (5,977 SNPs) and scores obtained from the whole-genome sequence data in 145 individuals. A. Correlation with homozygosity at alternate alleles grouped according to their allele frequency. B. Correlation with homozygosity at private alleles (young alleles) grouped according to their allele frequency. C. Correlation with marker homozygosity (counted at both reference and alternate alleles) as a function of minor allele frequency. D. Correlation with whole-genome homozygosity. The error bars represent the 95% confidence intervals.

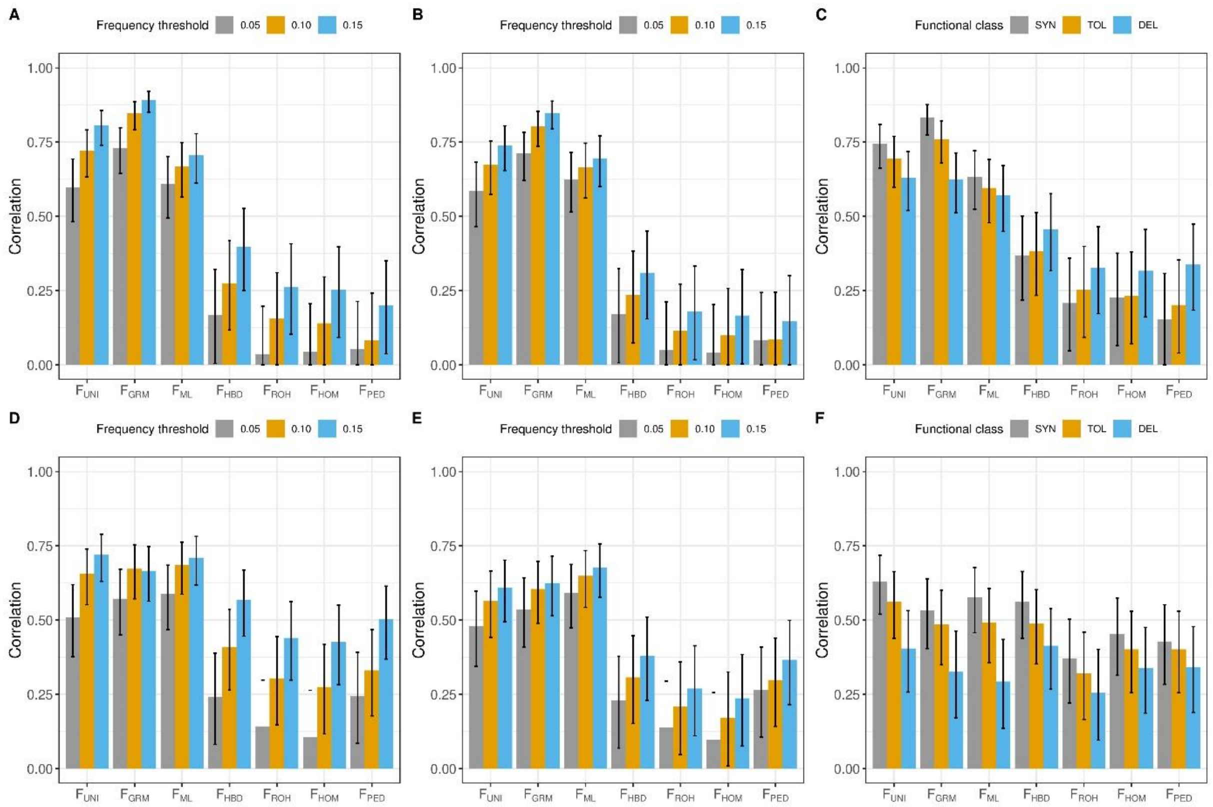

Supplementary Figure S20. Correlation coefficients between individual inbreeding measures estimated at lower marker density (5,977 SNPs) and homozygous mutation load (HML) computed using alternate (A, B, C) and private alternate (D, E, F) alleles from the whole-genome sequence data in 145 individuals. A and D. Correlation with HML estimated with allele frequency thresholds of 0.05, 0.10 and 0.15. B and E. Correlation with weighted HML estimated with allele frequency thresholds of 0.05, 0.10 and 0.15. C and F. Correlation with HML estimated with synonymous (SYN), tolerated (TOL) and deleterious (DEL) missense variants and using an allele frequency threshold of 0.15. The error bars represent the 95% confidence intervals and are truncated at 0.

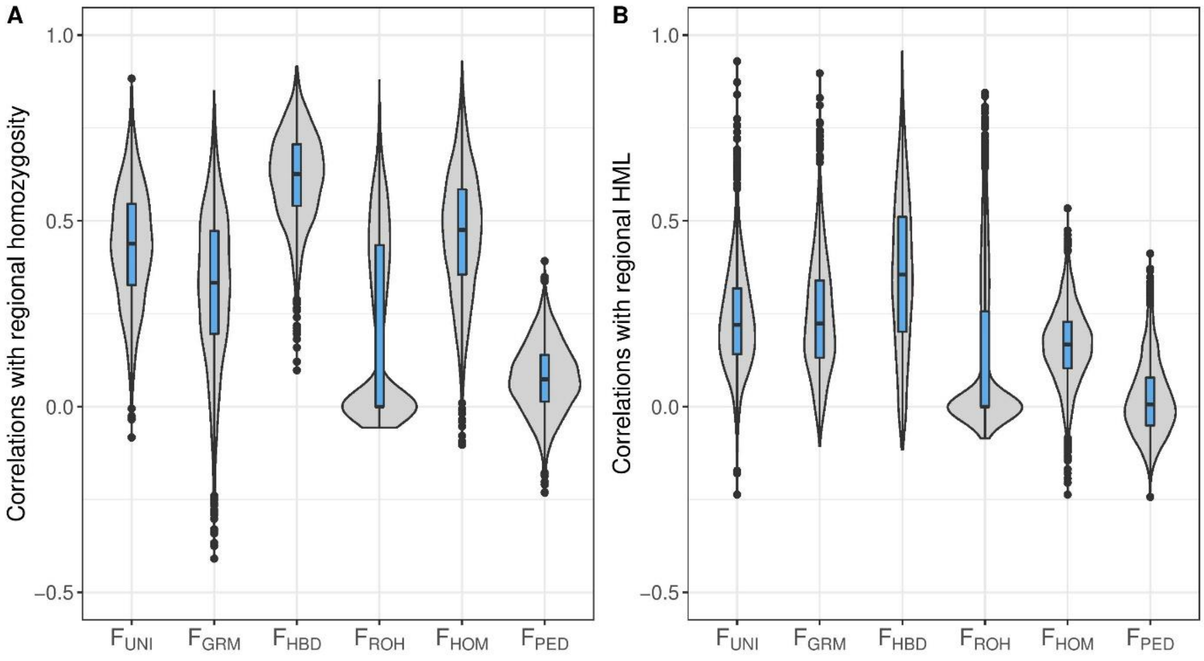

Supplementary Figure S21. Correlation coefficients between individual regional inbreeding measures and regional scores in 1 Mb windows computed from the whole-genome sequence data in 145 individuals. The regional inbreeding coefficients were estimated only with markers present among the 5,977 SNPs from the bovine low-density genotyping array. The correlations for approximately 2500 windows are presented as a violin plot combined with an inner boxplot. A. Correlation with regional homozygosity. B. Correlation with regional homozygous mutation load (HML). Note that when estimated inbreeding coefficients were constant for a window, a null correlation was obtained.

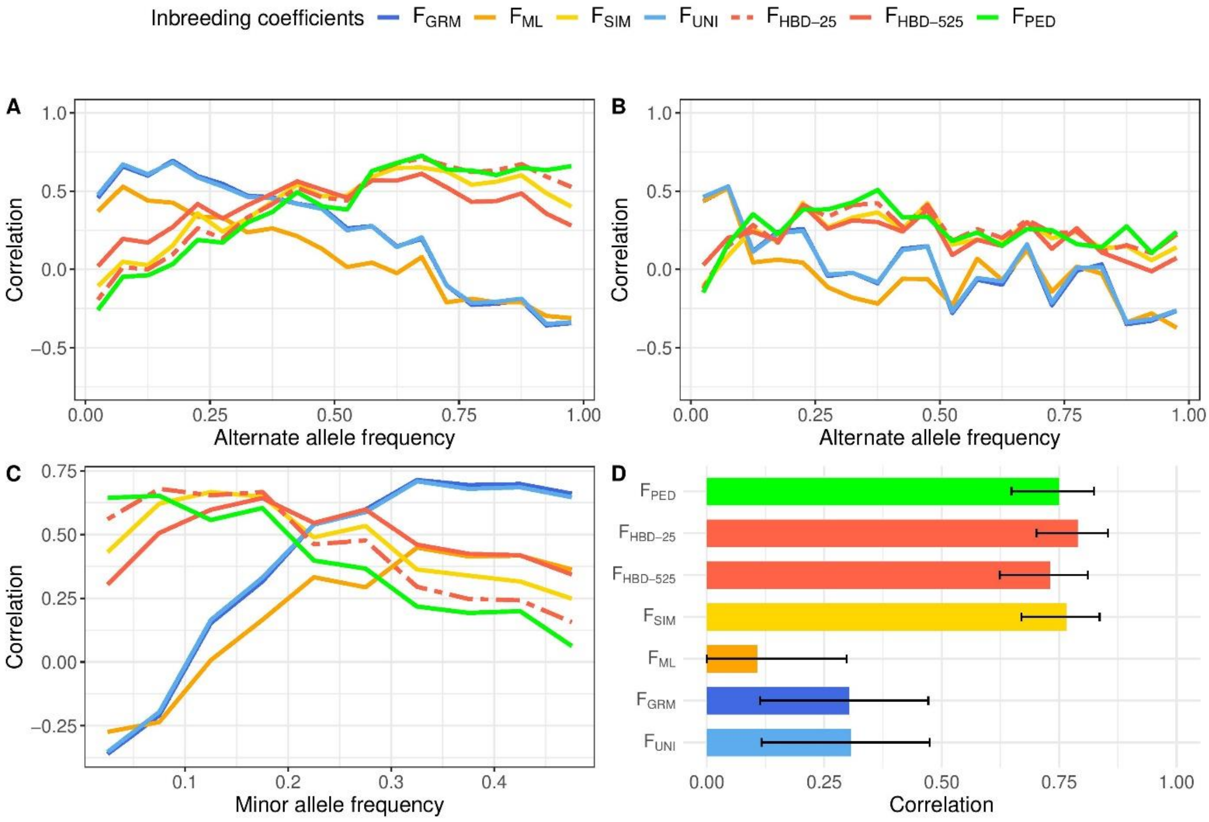

Supplementary Figure S22. Correlation coefficients between individual inbreeding measures predicted using parental genotypes at 37,675 SNPs and scores obtained from the whole-genome sequence data in their 100 offspring. A. Correlation with homozygosity at alternate alleles grouped according to their allele frequency. B. Correlation with homozygosity at private alleles (young alleles) grouped according to their allele frequency. C. Correlation with marker homozygosity (counted at both reference and alternate alleles) as a function of minor allele frequency. D. Correlation with whole-genome homozygosity. The error bars represent the 95% confidence intervals.

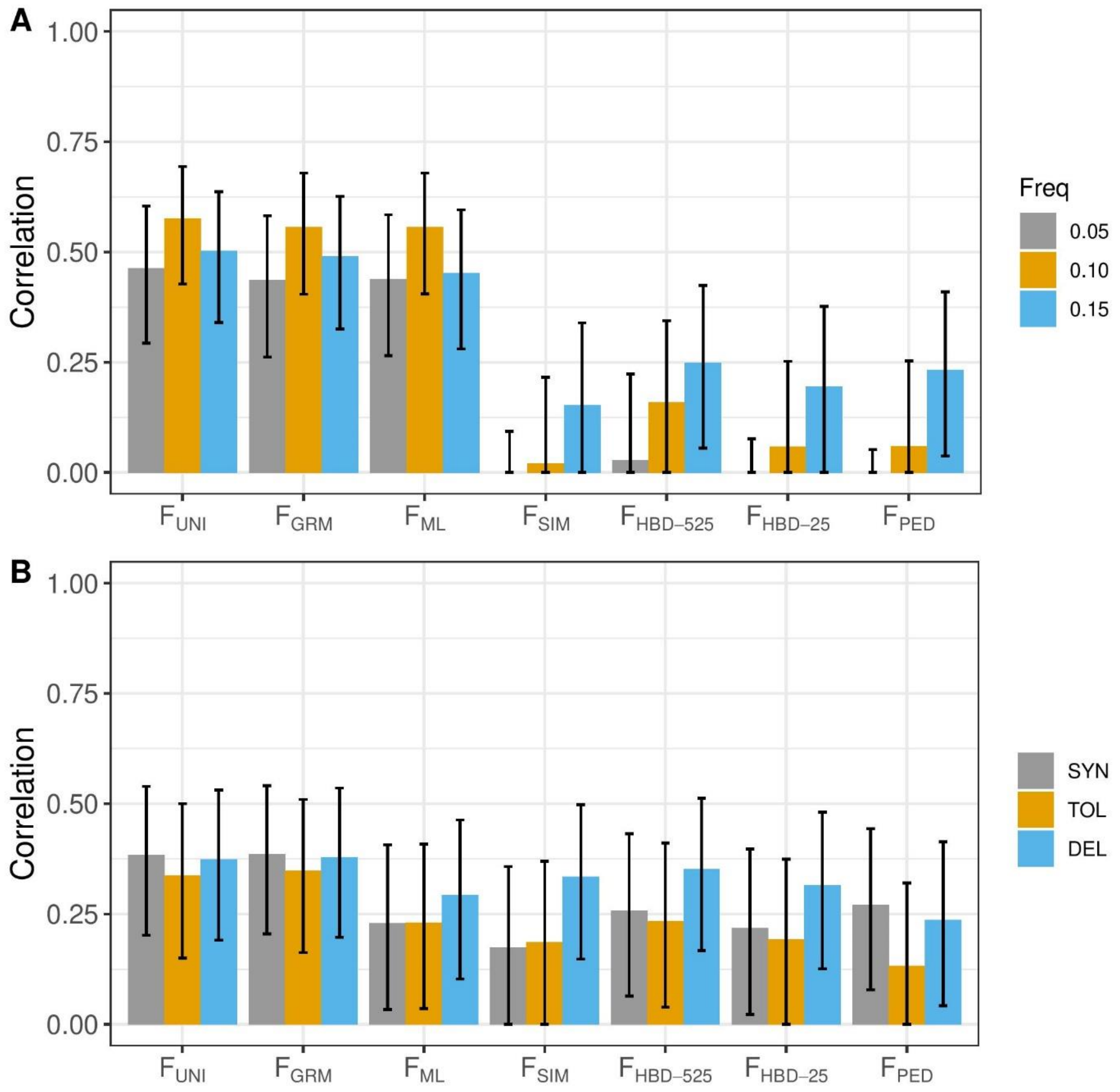

Supplementary Figure S23. Correlation coefficients between individual inbreeding measures predicted using parental genotypes at 37,675 SNPs and homozygous mutation load (HML) computed using private alleles from the whole-genome sequence data in their 100 offspring. A. Correlation with HML estimated with allele frequency thresholds of 0.05, 0.10 and 0.15. B. Correlation with HML estimated with synonymous (SYN), tolerated (TOL) and deleterious (DEL) missense variants and using an allele frequency threshold of 0.15. Note that negative values are not apparent. The error bars represent the 95% confidence intervals and are truncated at 0.

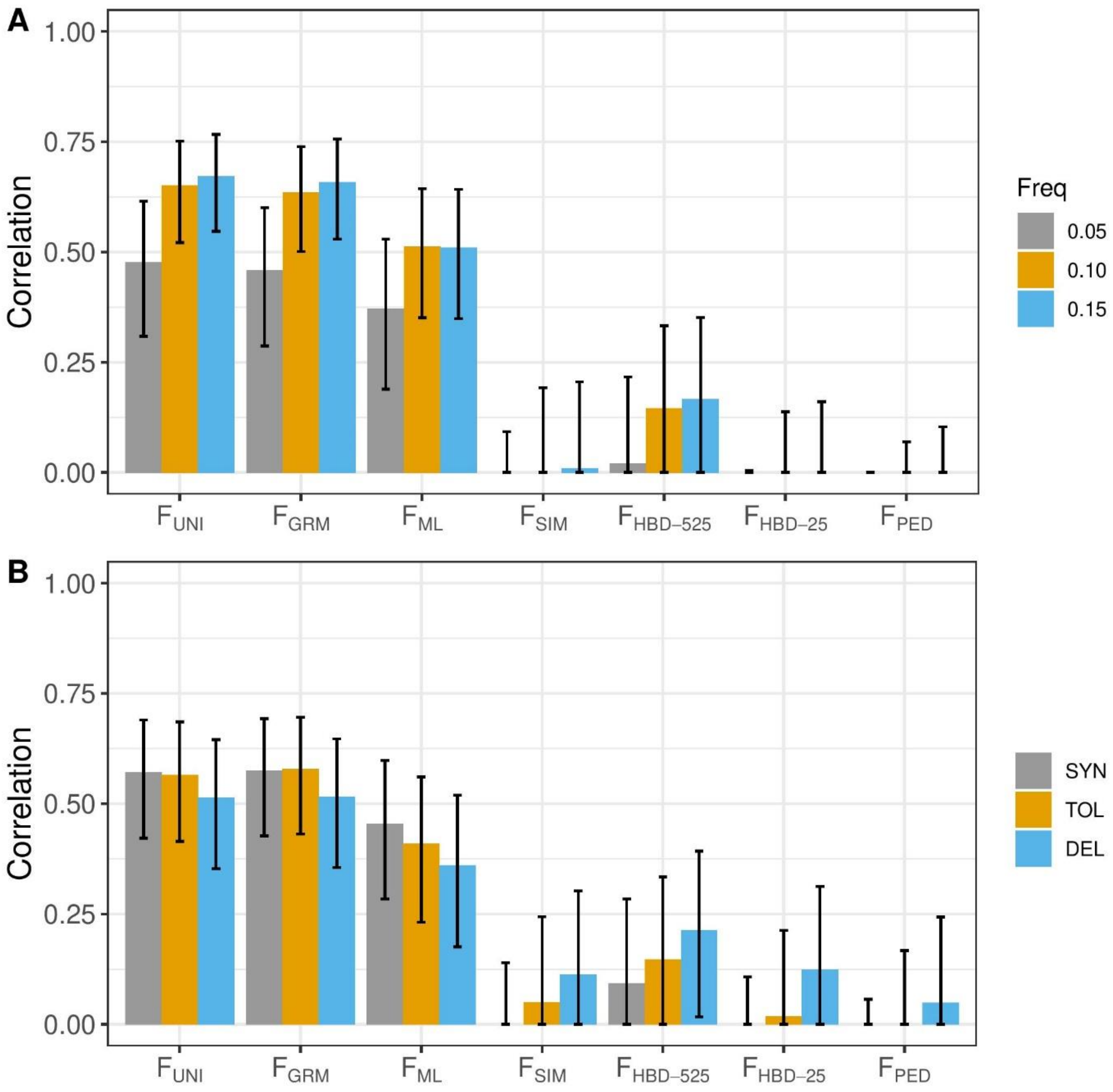

Supplementary Figure S24. Correlation coefficients between individual inbreeding measures predicted using parental genotypes at 37,675 SNPs and homozygous mutation load (HML) computed using alternate alleles from the whole-genome sequence data in their 100 offspring. A. Correlation with HML estimated with allele frequency thresholds of 0.05, 0.10 and 0.15. B. Correlation with HML estimated with synonymous (SYN), tolerated (TOL) and deleterious (DEL) missense variants and using an allele frequency threshold of 0.15. Note that negative values are not apparent. The error bars represent the 95% confidence intervals and are truncated at 0.
